# An Integrative Adapt Therapy for common mental health symptoms and adaptive stress amongst Rohingya, Chin, and Kachin refugees living in Malaysia: A randomized controlled trial

**DOI:** 10.1371/journal.pmed.1003073

**Published:** 2020-03-31

**Authors:** Alvin Kuowei Tay, Hau Khat Mung, Mohammad Abdul Awal Miah, Susheela Balasundaram, Peter Ventevogel, Mohammad Badrudduza, Sanjida Khan, Karen Morgan, Susan Rees, Mohammed Mohsin, Derrick Silove

**Affiliations:** 1 School of Psychiatry, Faculty of Medicine, University of New South Wales, Australia; 2 Perdana University-Centre for Global Health and Social Change (PU-GHSC), Selangor, Malaysia; 3 Perdana University-Centre for Research Excellence (PU-CRE), Selangor, Malaysia; 4 Health Unit, United Nations High Commissioner for Refugees (UNHCR), Kuala Lumpur, Malaysia; 5 Public Health Section/ Division of Programme Support & Management, United Nations High Commissioner for Refugees (UNHCR), Geneva, Switzerland; 6 Department of Psychology, Jagannath University, Dhaka, Bangladesh; 7 Perdana University-Royal College of Surgeons in Ireland (PU-RCSI) School of Medicine, Selangor, Malaysia; Johns Hopkins University Bloomberg School of Public Health, UNITED STATES

## Abstract

**Background:**

This randomised controlled trial (RCT) aims to compare 6-week posttreatment outcomes of an Integrative Adapt Therapy (IAT) to a Cognitive Behavioural Therapy (CBT) on common mental health symptoms and adaptive capacity amongst refugees from Myanmar. IAT is grounded on psychotherapeutic elements specific to the refugee experience.

**Methods and findings:**

We conducted a single-blind RCT (October 2017 –May 2019) with Chin (39.3%), Kachin (15.7%), and Rohingya (45%) refugees living in Kuala Lumpur, Malaysia. The trial included 170 participants receiving six 45-minute weekly sessions of IAT (97.6% retention, 4 lost to follow-up) and 161 receiving a multicomponent CBT also involving six 45-minute weekly sessions (96.8% retention, 5 lost to follow-up). Participants (mean age: 30.8 years, SD = 9.6) had experienced and/or witnessed an average 10.1 types (SD = 5.9, range = 1–27) of traumatic events. We applied a single-blind design in which independent assessors of pre- and posttreatment indices were masked in relation to participants’ treatment allocation status. Primary outcomes were symptom scores of Post Traumatic Stress Disorder (PTSD), Complex PTSD (CPTSD), Major Depressive Disorder (MDD), the 5 scales of the Adaptive Stress Index (ASI), and a measure of resilience (the Connor–Davidson Resilience Scale [CDRS]). Compared to CBT, an intention-to-treat analysis (*n* = 331) at 6-week posttreatment follow-up demonstrated greater reductions in the IAT arm for all common mental disorder (CMD) symptoms and ASI domains except for ASI-3 (injustice), as well as increases in the resilience scores. Adjusted average treatment effects assessing the differences in posttreatment scores between IAT and CBT (with baseline scores as covariates) were −0.08 (95% CI: −0.14 to −0.02, *p* = 0.012) for PTSD, −0.07 (95% CI: −0.14 to −0.01) for CPTSD, −0.07 for MDD (95% CI: −0.13 to −0.01, *p* = 0.025), 0.16 for CDRS (95% CI: 0.06–0.026, *p* ≤ 0.001), −0.12 (95% CI: −0.20 to −0.03, *p* ≤ 0.001) for ASI-1 (safety/security), −0.10 for ASI-2 (traumatic losses; 95% CI: −0.18 to −0.02, *p* = 0.02), −0.03 for ASI-3 (injustice; (95% CI: −0.11 to 0.06, *p* = 0.513), −0.12 for ASI-4 (role/identity disruptions; 95% CI: −0.21 to −0.04, *p* ≤ 0.001), and −0.18 for ASI-5 (existential meaning; 95% CI: −0.19 to −0.05, *p* ≤ 0.001). Compared to CBT, the IAT group had larger effect sizes for all indices (except for resilience) including PTSD (IAT, d = 0.93 versus CBT, d = 0.87), CPTSD (d = 1.27 versus d = 1.02), MDD (d = 1.4 versus d = 1.11), ASI-1 (d = 1.1 versus d = 0.85), ASI-2 (d = 0.81 versus d = 0.66), ASI-3 (d = 0.49 versus d = 0.42), ASI-4 (d = 0.86 versus d = 0.67), and ASI-5 (d = 0.72 versus d = 0.53). No adverse events were recorded for either therapy. Limitations include a possible allegiance effect (the authors inadvertently conveying disproportionate enthusiasm for IAT in training and supervision), cross-over effects (counsellors applying elements of one therapy in delivering the other), and the brief period of follow-up.

**Conclusions:**

Compared to CBT, IAT showed superiority in improving mental health symptoms and adaptative stress from baseline to 6-week posttreatment. The differences in scores between IAT and CBT were modest and future studies conducted by independent research teams need to confirm the findings.

**Trial registration:**

The study is registered under Australian New Zealand Clinical Trials Registry (ANZCTR) (http://www.anzctr.org.au/). The trial registration number is: ACTRN12617001452381

## Author summary

**Why was this study done?:**

- Integrative Adapt Therapy (IAT) is a theoretically guided approach based on the Adaptation and Development After Persecution and Trauma (ADAPT) model, specific to the refugee experience.
- The 5 pillars identified in the ADAPT model—(1) safety/security, (2) attachments, (3) justice, (4) role/identity disruptions, (5) existential meaning—may have universal resonance with refugees and displaced persons.
- This is the first randomised controlled trial (RCT) in which IAT is compared with a brief, culturally adapted 6 weekly 45-minute Cognitive Behavioural Therapy (CBT).

**What did the researchers do and find?:**

- Chin, Kachin, and Rohingya persons displaced to Malaysia were randomly assigned by an independent researcher to either the IAT or CBT control arm of the trial.
- Lay counsellors who received training in both IAT and CBT treated the participants according to their allocation status.
- Compared to CBT, IAT participants reported greater reductions in mental health symptoms, in all 5 adaptive stress areas (except the injustice domain), and a greater increase in resilience.

**What do the results mean?:**

- The findings suggest that IAT, delivered by lay counsellors, was an effective treatment for improving mental health, adaptive stress, and resilience amongst refugees.
- The difference in treatment effect between the 2 treatments on resilience is small.
- We note that our findings are limited to only 6-week follow-up and may not be generalisable to refugees in other settings.

## Introduction

In recent years, a range of brief psychological interventions have been developed for treating common mental health symptoms amongst refugees resettled in low-resource settings [1,2]. A task-shifting approach in which non-mental health professionals deliver the intervention after brief training make these interventions more feasible and cost effective [3]. Examples of generic interventions supported by systematic evidence include Problem Management Plus [4], Self-Help Plus [5], and the Common Elements Treatment Approach (CETA) [6].

To advance the field, it is important to ground interventions more specifically in the refugee experience, the focus of the novel Integrative Adapt Therapy (IAT) tested herein amongst Chin, Kachin, and Rohingya refugees exiled to Malaysia. IAT is based on the Adaptation and Development After Persecution and Trauma (ADAPT) model [7], which identifies 5 key psychosocial systems that support mental health in stable societies but are undermined by the refugee experience, including (I) safety/security, (II) interpersonal bonds and networks, (III) justice, (IV) identities and roles, and (V) existential meaning [8]. Examples of connections of these systems with mental health [9] include the impact of chronic or recurrent threats to safety and security (Pillar I of the ADAPT model) and the genesis and maintenance of fear and anxiety, which can lead to Post Traumatic Stress Disorder (PTSD) and related forms of clinical anxiety [10]; the cumulative effects of multiple traumatic losses and separations on bonds and networks (ADAPT Pillar II), leading to normative, and in some cases, prolonged grief reactions [11–13]; exposure to gross human rights violations (Pillar III: Justice), provoking anger, which, in some instances, can become dysfunctional, manifesting in ill-directed aggression [14,15]; loss of roles and identities (Pillar IV), which can provoke feelings of marginalisation and aimlessness, resulting in depression [13]; and disruption of systems of meaning in the social, cultural, political, spiritual, and religious domains (Pillar V), generating disruption of belief systems and values, leading to existential confusion, alienation, and depression [16]. Applying a recently developed measure, we have shown that the stressors associated with the ADAPT model influence multiple pathways involving past trauma and ongoing living difficulties leading to PTSD symptoms [10].

IAT therefore aims to improve mental health by promoting adaptive capacity and resilience [17–19]. For example, in treating a torture survivor [20], the counsellor may elect to focus on Pillar III, in which the emphasis is on addressing feelings of injustice and related symptoms of explosive anger. By understanding the genesis of these problems, the survivor becomes motivated to learn evidence-based techniques to manage their aggressive tendencies, thereby improving their sense of control, interpersonal relationships, and mental health [21]. Our comparator intervention, Cognitive Behavioural Therapy (CBT),[21] uses similar evidence-based strategies but omits the contextualisation of the therapy within the informing framework of the ADAPT model. The strategies used herein have been tested in interventions such as Problem Management Plus [4] and Self-Help Plus [5] and include psychoeducation, stress management, behavioural activation, cognitive reappraisal, and in vivo exposure.

Randomised controlled trials (RCTs) comparing 2 active psychotherapies are relatively rare in the refugee mental health and the wider general trauma field [22]. Quantitative differences in outcomes are likely to be small in such comparisons because of the elements common to both arms of the treatment, including the placebo effect, the encounter with an empathic counsellor, the general tendency for regression to the mean over time, and ceiling effects, that is, the inclusion of nonresponders in both arms [23]. Superiority of one treatment over the other therefore should be judged on relatively small differences in effect sizes and, more importantly, on the consistency in the pattern of differences across a range of relevant measures [23]. For that reason, we include symptoms of the comorbid common mental disorders (CMD) known to be relevant to refugees, including PTSD [13], Complex PTSD (CPTSD) [24, 25], Major Depressive Disorder (MDD) [26], Generalised Anxiety Disorder (GAD) [26], and Persistent Complex Bereavement Disorder (PCBD) [11,27].

For this first report testing the efficacy of IAT, we restrict ourselves to analysing outcome indices of key theoretical and clinical importance in the refugee field. We will present the findings of outcomes of more specialist or theoretical interest in a future report. Here, we examine the following hypotheses: (1) that both the IAT and the CBT conditions will produce significant improvements amongst refugees on all symptom measures at 6 weeks posttherapy, (2) that there will be a consistent pattern of superiority of IAT in improving a range of CMD symptoms, and (3) that IAT will show superiority over CBT in promoting adaptive capacity [28] and resilience. Given the strong interest in IAT by an increasing number of humanitarian agencies [21,29,30], we deemed it timely to report the 6-week posttreatment outcomes. We plan to report the 12-month outcomes by late 2020 when data for that longer follow-up are complete and analysed.

## Methods

### Ethical approval

This study was approved by the Human Research Ethics Committee (HREC) of the University of New South Wales and the Institutional Review Board, Perdana University-Royal College of Surgeons in Ireland (PURCSI) School of Medicine, Malaysia.

### Study design and participants

This single-blind two-armed parallel RCT was conducted between October 2017 and May 2019 amongst refugees from the Chin, Kachin, and Rohingya communities who had fled persecution in Myanmar (S1 Text). All participants were registered with the United Nations High Commissioner for Refugees (UNHCR) in Malaysia. These communities continue to live under conditions of insecurity, risking arrest, detention, and deportation; many experience extreme poverty and face difficulties obtaining education and access to healthcare and other services, including in mental health.

Participants meeting inclusion criteria were recruited serially from a clustered, multistage epidemiological study (September—December 2018) conducted amongst the 3 ethnic groups concentrated in and around Kuala Lumpur, the capital of Malaysia. Inclusion criteria were (a) presence of at least one of the designated CMDs, that is, current PTSD/CPTSD, MDD, GAD, and PCBD [31]; (b) witnessed or experienced at least one traumatic event related to mass conflict; and (c) endorsed at least one ADAPT-related stressor on each scale of the relevant measure (see hereunder). Exclusion criteria were age lower than 18 years, overt evidence of intellectual disability or cognitive impairment, or manifestations of psychosis (as assessed using WHO mhGAP protocol) [32]. Refugees who met criteria and consented to participate were randomly assigned to either IAT or CBT according to a 1:1 ratio determined by a computer-generated randomisation sequence managed by an off-site research assistant.

This study is designed and reported according to the Consolidated Standards of Reporting Trials (S2 Text) guidelines. All participants were assessed at baseline, at 6-week posttreatment, and are being followed up at 12 months. Consistent with CONSORT, masking was applied to the assessment team, data manager, and statistician in relation to participant treatment arm allocation and to baseline and follow-up assessments for the treatment team.

In the course of 3 parallel epidemiological studies conducted with the Chin, Kachin, and Rohingya communities (results to be reported elsewhere), a random sample of 1,103 participants were assessed for eligibility for inclusion in the intervention study. Of these, 618 did not meet inclusion criteria, 100 left Malaysia prior to the intervention study to be resettled in a third country, and 54 relocated at distant locations within Malaysia for work-related reasons.

**Fig 1:**
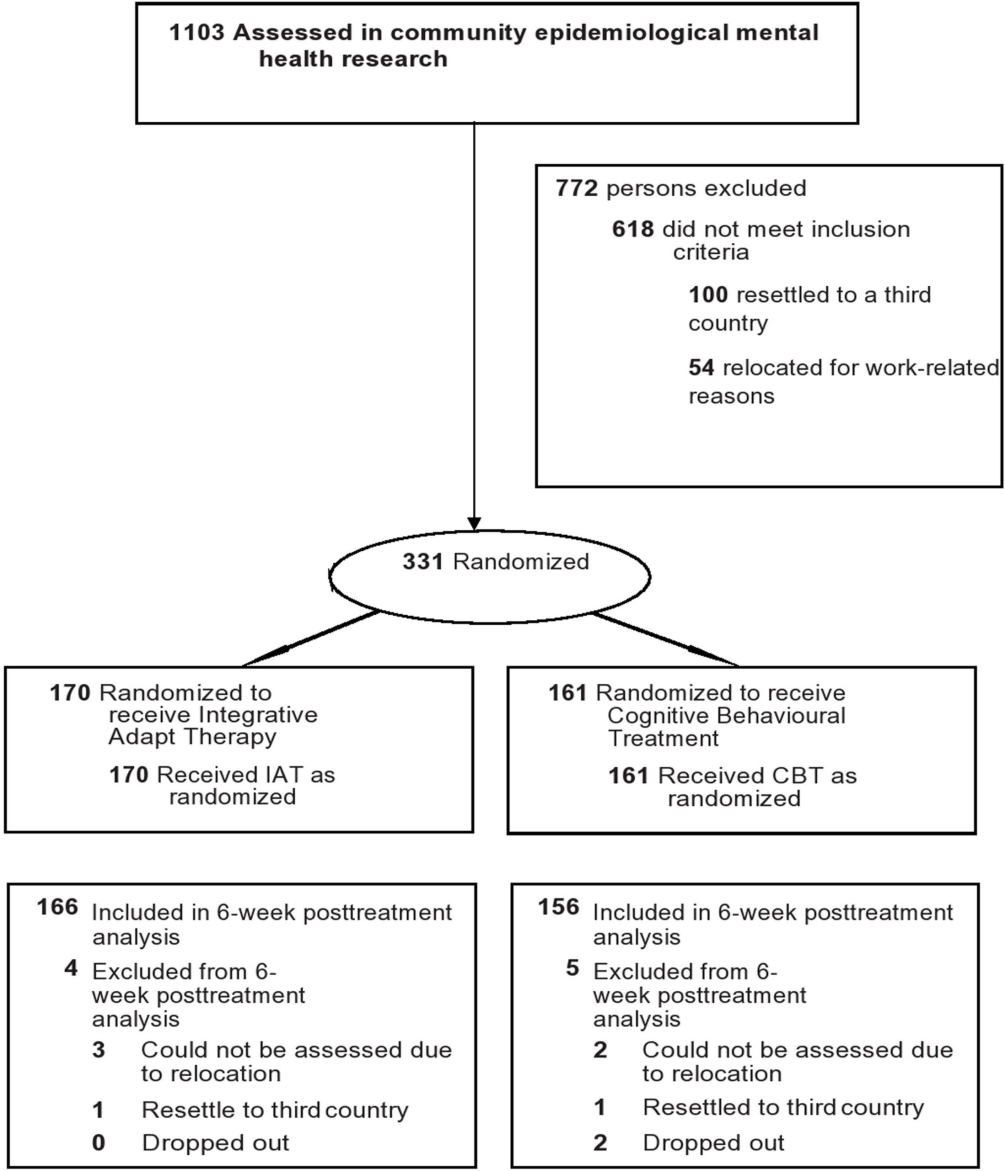
Flow Chart of participants through phases of a randomized trial comparing IAT versus CBT amongst trauma-affected refugees from Myanmar living in Malaysia. CBT, cognitive behavioural therapy; IAT, integrative adaptive therapy.

Only 2 participants intentionally discontinued therapy, both in the CBT arm. Four in the IAT arm and three in the CBT arm could not be contacted during the course of therapy because they had relocated to distant parts of the country or overseas (Fig 1). All participants provided written consent prior to participation in accordance with the ethical requirements of respective institutions.

### Intervention

Table 1 describes the theoretical background and treatment strategies relevant to both IAT and CBT.

**Table 1:** Theoretical background and treatment strategies for IAT and CBT.

**IAT**
45-minute, 6-weekly sessions
Delivered by trained lay counsellors
IAT is skills-based and includes 7 strategies (outlined hereunder)
The content of the therapy in which these techniques are applied draws specifically on the ADAPT model in which each pillar—and its psychosocial impact—are considered serially in relation to the individual’s personal experiences
IAT focuses explicitly on the 5 psychosocial pillars of the ADAPT model: safety/security, losses and separation, injustice, role and identity disruptions, and existential meaning
IAT makes more explicit an ecological perspective (ADAPT) in tracing in a thematic manner the major disruptions in psychosocial support systems that the refugee has experienced through his or her trajectory of displacement. In each case, the emphasis may differ depending on the issues identified by each individual
**IAT Treatment Strategies**
**1. Psychoeducation**
Highlight the common experiences of all refugees
Link program to the ADAPT model
Link the refugee experience to the 5 ADAPT domains
Focus on building resilience and adaptive capacity to manage distress associated with the core refugee challenges, avoiding labelling mental disorders
Provide information about the program (duration, benefits, expectations)
**2. Trauma narrative/modified exposure**
Identity significant stressful life events and narrate these (traumatic) experiences in a coherent and chronological manner
Link each set of events to each of the 5 ADAPT pillars where appropriate, including safety/security, attachments, justice, role transition/identity, and meaning
Normalize feelings of fear and anxiety
Understand the link between past trauma and present and future challenges, linking these events to the ADAPT model
**3. Problem solving**
Identity at least 3 problems according to each ADAPT domain and focus on the one(s) most preoccupied with
Explore the underlying feelings of distress and reactions to each problem
Explore coping methods, strategies, and any existing barriers and/or perpetuating factors
Brainstorm solutions and adopt a solution
Commit to trying the solution in a step by step manner
**4. Stress management**
Apply strategies to manage stress: controlled breathing, progressive muscle relaxation incorporating locally salient metaphors and analogies
**5. Emotion regulation**
Apply emotion regulation strategies to deal with and build tolerance for distress associated with the disrupted ADAPT pillars
Identify and label emotions/feelings using visually salient pictorial aids
Normalize feelings of distress
Accepting emotions/feelings and letting go without judgement
Distancing self from unpleasant feelings
**6. Cognitive reappraisal**
Understand thoughts, feelings, and behaviour and how these are connected
Identify and challenge unhelpful, negative thoughts/beliefs according to the experiences arising from the ADAPT pillars
Understand and overcome gaps between expectations and reality with an emphasis on change in role transition and identity before and postmigration
**7. Meaning making**
Accepting the reality, recognizing and appreciating all small things in life
Give hope
Find meaning in life (what is worth living for, e.g., goals, dreams)
Committing to goals and a life worth living
**CBT**
45-minute, 6-weekly sessions
Delivered by trained lay counsellors
CBT includes 6 strategies—drawn from WHO PM and with an additional component of cognitive reappraisal
The strategies are delivered sequentially over 6 sessions, each session is built on the previously learnt techniques
CBT is primarily aimed at addressing maladaptive cognitive and behavioural patterns of responding to adversity, trauma
The content of the therapy varies, with each session focusing on learning new coping skills and building on previously learnt skills.
**CBT Treatment Sessions**
**Session 1: Psychoeducation**
Introduction and confidentiality
What is CBT
Understanding how adversity impacts on mental health
Managing stress
Ending session
**Session 2: Problem-solving, managing stress**
General review
Managing problems
Managing stress
Ending session
**Session 3: Problem-solving, managing stress, and behavioural activation**
General review
Managing problems
Get going, keep going
Managing stress
Ending the session
**Session 4: Problem-solving, managing stress, and behavioural activation, strengthening social support**
General review
Managing problems
Get going, keep going
Strengthening social support
Managing stress
Ending the session
**Session 5: Problem-solving, managing stress, and behavioural activation, strengthening social support, cognitive reappraisal**
General review
Managing problems
Get going, keep going
Strengthening social support
Managing stress
Cognitive reappraisal
Ending the session
**6. Ending treatment**
General review
Staying well
Imagining how to help others
Looking to the future
Ending the program

Adapted from Tay, A. K. et al. Theoretical background, first stage development and adaptation of a novel IAT for refugees. Epidemiology and Psychiatric Sciences, 1–8, doi:10.1017/S2045796019000416 (2019)

**Abbreviations:** ADAPT, Adaptation and Development After Persecution and Trauma; CBT, Cognitive Behavioural Therapy; IAT, Integrative Adapt Therapy

#### IAT

A detailed description of the theoretical foundations and cultural adaptation of IAT has been published elsewhere [20].

The IAT program involved 6 weekly 45-minute sessions. As indicated, IAT is grounded on the 5 psychosocial pillars of the ADAPT model [20]. Refugees participating in IAT are encouraged to reflect on past and ongoing experiences related to the disruptions of the psychosocial foundations of their societies, their families, and their personal lives as they transitioned through the trajectory of mass conflict, upheaval, displacement, flight, and resettlement. Connections are made between these experiences, the meaning to the person, and symptoms and maladaptive behaviours that may be causing personal or interpersonal difficulties. The strategies then offered for dealing with these issues are framed in a manner that ensures their integration within the broader ADAPT model. These strategies include psychoeducation, trauma narrative/in vivo exposure, problem solving, stress management, emotion regulation, cognitive reappraisal, and meaning making.

#### CBT

The CBT condition involved 6 weekly 45-minute sessions and included 6 core treatment strategies: psychoeducation, stress management, problem solving, behavioural activation, cognitive reappraisal, and strengthening social support, based on existing evidence of the effectiveness of these element and their suitability for application by lay counsellors based on the principle of task shifting [4].

Each strategy was introduced sequentially over the course of 6 sessions and each session was designed to build on the previously learnt techniques. Participants were given homework practice to enhance their mastery of the skills taught. Although the same techniques were used in both therapies, the major difference was that the overarching ADAPT framework was not included in the CBT arm. Instead, the treatment was presented as an intervention to manage stress, current problems, and interactions with others. Where appropriate, consideration of past traumatic events was inculcated in the procedure. For example, in the session focusing on cognitive reappraisal, a refugee who reported feelings of guilt and shame following sexual assault was taught cognitive reappraisal techniques to address these maladaptive thoughts and feelings. A fuller account of the distinctive features of IAT compared to other CBT-derived treatments are detailed in our background papers [20,21].

### Development, adaptation, and piloting of interventions and manuals

We have previously documented the systematic process of developing, adapting, and piloting IAT amongst refugees in Malaysia [20] and amongst Rohingya refugees displaced to Cox’s Bazaar, Bangladesh [30].

Parallel but separate procedures were applied in developing and refining both the IAT and CBT interventions. The systematic process of cultural adaptation of both therapies was guided by the framework of Bernal and Saez-Sanriago [33] and based on an extensive review of cultural idioms of distress and local terminologies for mental health symptoms [34,35]. Parallel draft manuals and associated materials were translated into the relevant dialects with the assistance of bilingual mental health professionals. An iterative process of qualitative research assisted in refining the accuracy and cultural appropriateness of the translations. For each intervention, feedback was sought from focus groups and interviews with key informants from each of the 3 ethnic groups, involving a range of community members and clinicians experienced in refugee mental health. All materials were translated into Bangla and Burmese, the former being the closest written language to the Rohingya dialect, which lacks a standard written format. Treatment manuals were standardized to ensure equivalence in the length of sessions (6 × 1 weekly 45-minute sessions), language (simplifying and replacing technical terms with colloquial expressions), delivery, and administration, while maintaining the key elements of each approach. Where appropriate, manuals were embellished with metaphors including materials of specific cultural relevance, including stories, idioms and symbols to facilitate comprehension of the content.

### Personnel selection, training, competency assessment, and supervision

Details of training, supervision, and competency are reported elsewhere [20,21].

The intervention team consisted of 28 lay counsellors (8 from the Chin and Kachin communities and 12 from the Rohingya communities, ensuring a gender balance for each group). All treatment sessions were conducted by lay counsellors either at community offices or at the residences of the participants. Each counsellor conducted both IAT and CBT. Eight days were devoted to training in IAT and the same period for CBT, the 2 programs being taught separately, using 2 manuals—one for IAT and one drawing on WHO PM+ but with appropriate modifications to ensure comparability in therapeutic time and other generic details with the delivery of IAT [4]. Half of the counsellors received the 2 sets of training in one sequence, the other in the reverse order. In both trainings provided by the research team (MAAM, SK, AKT, HKM), care was taken not to imply a preference for one treatment over the other.

Training covered a range of topics including general mental health and psychosocial issues relevant to refugees, cultural notions of mental health and culturally relevant idioms of distress, generic counselling skills (attentive listening, rephrasing, empathy), the foundations of ADAPT and CBT therapy in sequence, systematic study of treatment modules of IAT and CBT, ethical principles in psychotherapy with vulnerable and refugee persons, and role play and other simulations of interventions.

Trainees then progressed to 8 weeks of field practice with members of the respective communities meeting selection criteria, followed by 6 months of supervised implementation prior to commencement of the trial [21] in which 20 assessments and 20 in vivo sessions of each therapy were observed and rated by supervisors (HKM, MB, MAAM, SK, AKT).

Each counsellor was assessed for their competency in basic counselling and in applying the treatment strategies specific to both CBT and IAT. Twenty-two of the twenty-eight trained counsellors scored a 2 (pass) on all items used to evaluate both basic counselling and level of fidelity to each treatment approach (S3 Text). 240 sessions (120 IAT sessions +120 CBT sessions) were observed by Burmese/Rohingya-speaking clinical supervisors with postgraduate qualifications and extensive practice in clinical psychology (MAAM, SK, AKT) and/or social work (HKM, MB). In this process, 108 (90%) and 110 (91.7%) sessions, respectively, were rated as satisfactory or pass. The 6 counsellors who failed to achieve a satisfactory score on all items in the first round of assessments were given additional 1:1 support in further practice in order to enhance their skills, and on reassessment, each achieved a pass on at least 80% of the rated items.

### Treatment fidelity

Treatment fidelity for all counsellors in both modalities of therapy was assessed by 2 independent assessors, each trained in either IAT or CBT. Separate fidelity ratings and clinical skills assessments were developed for IAT and CBT. Assessors directly observed a 10% random selection of each counsellor’s sessions (10 sessions per counsellor), rating them on a checklist and providing an additional narrative commentary regarding their competency, accuracy, and consistency in delivery of the relevant therapeutic modality. The summary IAT assessment was based on a summation of both general counselling skills and IAT-specific strategies [30] (rated on a 3-point scale: 1 = failed to deliver the technique, 2 = delivered some but not all the elements of a technique, 3 = delivered all elements of a technique). For CBT, we drew on the steps outlined in each of the 5 sessions included in the PM+ approach with an additional sixth session that included cognitive reappraisal. The criteria used to assess competency and fidelity in CBT were based on whether each step of a session is delivered to the satisfactory level. For example, a trainee is rated 1 if he/she failed to deliver the session in all its elements, 2 if some (but not all) of the steps were delivered, and 3, if all prescribed steps were delivered to the satisfactory level. To proceed to the final part-II assessment, the trainee needed to score at least 2 (pass) for 80% of the items in both the general counselling skills and IAT-specific or CBT-based skills. All trainees were required to pass both stages of assessment. Further training and supervision were provided for counsellors who failed any component of the assessment until a satisfactory rating was achieved.

### Measures

#### Assessment team

A team of 5 research assistants (each receiving 5 days of training in the assessment protocol) conducted baseline and follow-up assessments independently of the intervention team. A standard of Inter-Rater Reliability (IRR) of assessments (90% concordance in assigning PTSD, CPTSD, MDD, GAD, PCBD cases), applied previously [31], was required prior to the team member being certified to undertake definitive assessments. We repeated the IRR assessments until the expected concordance was achieved.

Suicide risk was assessed using the screening item of the Refugee Mental Health Assessment Package (RMHAP) depression module [31], supplemented by the modified suicide module of the mhGAP Humanitarian Intervention Guide (mhGAP-HIG) [32,36]. Participants are categorised as no risk (a score 1), low (a score of 2 ‘a little’), moderate (a score of 3 ‘quite a lot’), or high suicide risk (a score of 4 ‘extremely’) based on their responses on the screening item. The management plan for low-risk persons involved weekly monitoring, a safety plan, and removal of access to harmful or lethal methods; for moderate-risk, referrals are made to local psychiatric services and a family member or a trusted person is informed with a safety plan in place; for high-risk persons, in addition to the required steps as outlined above, an emergency protocol is implemented with 24/7 monitoring with possible hospital admission. Participants deemed high-risk would be excluded from the trial. The counsellors are trained in the safety protocol and are required to consult their clinical supervisors (clinical psychologists) (MAAM, SK) when such cases arise prior to implementing an action plan.

### Primary outcome measures

The RMHAP (S2 Table) [31] includes indices assessing trauma exposure, postmigration living difficulties, full symptoms of all CMDs included, and the ASI [28], the latter recording stress arising from erosion of the 5 ADAPT pillars consisting of five scales. The core modules of the RMHAP—those assessing CMDs and the ASI have been adapted and tested extensively in refugee groups [28, 31].

We used the RMHAP to assess full symptoms of PTSD, CPTSD, MDD, GAD, and PCBD based on the Diagnostic and Statistical Manual for Mental Disorders 5th edition (DSM-5) criteria with the exception of CPTSD, which was based on the International Classification of Diseases 11th edition (ICD-11) [37]. Symptoms are rated on a 4-point scale based on how frequently they are experienced (1 = not at all, 2 = a little bit, 3 = quite a lot, 4 = extremely); the 2 highest frequency categories are regarded as indicating a clinically significant endorsement of the symptom. In all interviews, participants are required to complete the full symptom list for each disorder without skip rules being applied. Responses were recorded directly on an electronic tablet to reduce errors in transcription and ensure rapid access to the data. Past psychometric analyses of both categorical and dimensional measures of the 5 CMDs included [31] each yielded evidence of sound internal consistency, test-retest reliability, and concurrent validity of the relevant diagnostic constructs in comparison with established gold standard diagnostic assessments [38]. A mean score for each CMD was calculated by adding the individual’s frequency responses (1–4) for all symptoms of each diagnostic category and these scores were used in pre- and posttreatment comparisons.

### Resilience

The CDRS [39] consists of 25 items assessing 5 resiliency factors: (1) personal competence, high standards, and tenacity; (2) trust in one’s instincts, tolerance of negative affect, and strengthening effects of stress; (3) positive acceptance of change and secure relationships; (4) control; and (5) spiritual influences. The measure has been widely used in trauma-affected populations, including amongst refugees, and past psychometric studies of the instrument have yielded high levels of internal consistency, test-retest reliability, and convergent validity [40, 41]. Items are scored on a 5-point Likert scale that ranges from ‘not true at all’ (scored 0) to ‘true nearly all the time’ (scored 4). Total scores range from 0 to 100; a mean score was calculated based on all items, with higher scores reflecting greater resilience.

### ASI

The ASI [28] comprises 5 scales, each derived from empirical testing of stressors arising from the erosion of corresponding ADAPT pillars: ASI-1 (12 items assessing safety and security), ASI-2 (14 items assessing traumatic losses and separations), ASI-3 (13 items assessing injustice), ASI-4 (11 items assessing role and identity disruptions), ASI-5 (14 items assessing existential meaning). Each item is scored on a 4-point Likert scale (0 = not at all, 1 = a little, 2 = quite a lot, 3 = extremely). A mean score is calculated for each ASI scale based on the aggregate of relevant item scores. The ASI has been subjected to extensive psychometric testing across several refugee groups [28].

### Secondary outcome measures

Symptoms of GAD and PCBD were assessed using the relevant modules of the RMHAP [27,31], as indicated above. A mean score was calculated for each diagnostic construct and used in pre- and posttreatment outcome analyses.

Assessments were made for suicidal and homicidal tendencies in the first session of both IAT and CBT. All counsellors were trained to undertake a systematic evaluation of risk assessment including completing a safety protocol comprising awareness raising, detection, normalisation, and referral (as indicated above). We did not include an assessment of substance use because we only identified a small minority (<0.2%) in our preceding epidemiological surveys who acknowledged a problem with alcohol and/or substance use.

### Statistical analyses

The International Society for Traumatic Stress Studies (ISTSS) has provided guidance concerning the magnitude of differences in outcome measures regarded as clinically meaningful in comparing one active psychotherapy over another in the trauma field [42]. Based on this guide, we estimated that a minimum of 150 participants were needed in each arm to achieve a moderate effect size of 0.50 and a design effect of 1.5, based on 80% power and a two-tailed 5% significance level. This calculation assumed an attrition rate of 50% to allow for the known pattern of ongoing resettlement to third countries that could result in unavoidable attrition over the follow-up period.

All analyses were conducted in STATA version 14. We applied an intention-to-treat analysis in modelling treatment effects over time, based on within-person changes in primary (PTSD, CPTSD, MDD, resilience, ASI domains) and secondary (GAD, PCBD) outcomes using mean scores. All variables were treated as continuous. For this study, we focused on the relevant outcomes of CMDs, adaptive capacity, and resilience, as these core indices are of key theoretical and empirical interest to the field of refugee mental health. For logistic and economic reasons, we adjusted our initial follow-up protocol to 6-week and (ongoing) one year posttreatment.

Initial unadjusted analyses were used to examine pre-post changes in outcomes within each arm as well as differences in baseline and posttreatment scores (at 6-week follow-up) between the IAT and CBT arms. To assess average treatment effects, we applied a random effects model in which assessment points (0 = baseline, 1 = follow-up) and counsellor ID were specified as random effects to account for within-person correlation effects across time and between-person correlation effects by counsellor.

Less than 1% of data were missing for both treatment and control groups for all sociodemographic and outcome variables at baseline and follow-up so that the application of imputation strategies was not required.

Prior to assessing adjusted models, we used *t* tests for continuous variables and x^2^ tests for categorical variables to test baseline differences in sociodemographic variables (sex, ethnicity, marital status, education, employment status) that might confound treatment outcomes. None of these variables were found to exert significant effects. For each adjusted model, we estimated the average treatment effect at follow-up by including the baseline score of each outcome as a covariate. We report adjusted mean differences at baseline and at 6-week follow-up indicating the significance level based on a two-sided *P <* 0.05 value and 95% confidence intervals.

Cohen’s *d* effect sizes were calculated by dividing the difference in model-adjusted average treatment effect between treatment and control by the pooled standard deviation of each relevant outcome index at baseline. Following an established approach [43], we calculated the effect size of each outcome based on the adjusted mean difference in which the baseline standard deviation of each outcome was used for the pooled participants from both arms, based on the assumption of constancy in baseline variance across time (over the 6-week follow-up). We followed the convention in which effect sizes of 0.2 are considered small, 0.5 medium, and 0.8 or above, large [44].

## Results

Fig 1 shows the flow of participants through the trial. Participants retained at 6-week follow-up included 166 in the IAT group (97.6%; 166 out 170) and 156 (96.8%; 156 out of 161) in the CBT group. The small number lost to follow-up had relocated to an unknown address or had resettled in a third country. Overall, participants attended a mean 5.5 sessions (SD = 1.4) involving an average of 48 minutes per session, with no significant differences in the number of sessions attended or the length of sessions across the 2 arms.

The sociodemographic characteristics of participants are reported in Table 2. There were no baseline differences in sociodemographic characteristics across the 3 ethnic groups or 2 treatment arms (Table 2). The sample consisted of Rohingya (45%), Chin (39.3%), and Kachin (15.7%) refugees. The mean age of the whole sample of 331 participants was 30.8 years (SD = 9.6). Almost three-quarters were men (71.9%), and over half were married (64%). Two-thirds of all participants had completed primary school education (68.2%), and a minority had graduated from secondary school (21.8%). Most were employed (86.7%) in a range of settings, including restaurants, construction sites, factories, and on rubber plantations. Participants had experienced or witnessed an average 10.1 types (SD = 5.9, range = 1–27) of traumatic events including exposure to conflict, persecution, physical assault, traumatic losses and separations, and poor physical health with no access to medical care.

**Table 2:** Demographic characteristics based on the full sample (*n* = 331).

Sociodemographic characteristics	Number (%)			x^2^ /t test *P* value
	Total (*n* = 331)	IAT (*n* = 170)	CBT (*n* = 161)	
**Sex**				
**Male**	238 (71.9)	124 (72.9)	114 (70.8)	
**Female**	93 (28.1)	46 (27.1)	47 (29.2)	*x*^*2*^(1) = 0.12, *P =* 0.73
**Ethnicity**				
**Rohingya**	149 (45)	76 (44.7)	73 (45.3)	
**Chin**	130 (39.3)	66 (38.8)	64 (39.8)	
**Kachin**	52 (15.7)	28 (16.5)	24 (14.9)	*x*^*2*^(3) = 1.94, *P =* 0.38
**Marital status**				
**Single**	105 (31.7)	56 (32.9)	49 (30.4)	
**Married/partnered**	212 (64)	107 (62.9)	105 (65.2)	
**Widowed**	7 (2.1)	3 (1.8)	4 (2.5)	
**Separated**	7 (2.1)	4 (2.4)	3 (1.9)	*x*^*2*^(3) = 2.29, *P =* 0.52
**Highest level of education completed**				
**None**	9 (2.7)	2 (1.2)	7 (4.3)	
**Primary school**	231 (69.8)	116 (68.2)	106 (65.8)	
**Secondary school**	72 (21.8)	34 (20)	38 (23.6)	
**University/college**	10 (3)	18 (10.6)	10 (6.2)	*x*^*2*^(3) = 2.42, *P =* 0.49
**Employment status**				
**Employed**	287 (86.7)	149 (87.6)	138 (85.7)	
**Unemployed**	44 (13.3)	21 (12.4)	23 (14.3)	*x*^*2*^(1) = 0.12, *P =* 0.73
**Age, mean (SD), range**	30.8 (9.6), 18–70	31.2 (9.9), 18–69	30.3 (9.3), 18–70	t(318) = 0.90, *P =* 0.37
**Number of traumatic events (experienced/witnessed), mean (SD), range**	10.1 (5.9), 1–27	10.5 (5.7), 1–26)	10.8 (5.5), 1–27	t(318) = −0.55, *P =* 0.65

**Abbreviations:** CBT, Cognitive Behavioural Therapy; IAT, Integrative Adapt Therapy

Baseline assessments were completed by 170 participants in the IAT and 161 in the CBT arms, respectively. There were no significant differences in baseline indices between the IAT and CBT arms (Table 3).

**Table 3:** Unadjusted and adjusted models examining average treatment effects within and between the IAT (*n* = 170) and CBT (*n* = 161) arms at baseline and 6-week follow-up (*n* = 331).

Outcomes	Unadjusted models					Adjusted models^a^	
	IAT (*n* = 170)		CBT (*n* = 161)		IAT versus CBT *P* value	Adjusted mean difference (95% CI)	IAT versus CBT *P* value
	*N*	Mean (95% CI)	*N*	Mean (95% CI)			
**Primary outcomes**							
**PTSD score**							
Pretreatment	170	1.66 (1.58–1.74)	161	1.64 (1.56–1.72)		0.01(−0.06–0.07)	
Posttreatment	166	1.26 (1.21–1.31)*↓	156	1.29 (1.25–1.33)*↓	<0.001	−0.08 (−0.14 to −0.02)	0.012
**Complex PTSD score**							
Pretreatment	170	1.67 (1.59–1.75)	161	1.65 (1.57–1.73)		0.01 (−0.06 to 0.07)	
Posttreatment	166	1.15 (1.11–1.19)*↓	156	1.21 (1.16–1.26)*↓	<0.001	−0.07 (−0.14 to −0.01)	0.025
**MDD score**							
Pretreatment	170	1.82 (1.74–1.09)	161	1.82 (1.74–1.9)		−0.001 (−0.06 to 0.06)	
Posttreatment	166	1.27 (1.24–1.30)*↓	156	1.33 (1.28–1.38)*↓	<0.001	−0.07 (−0.13 to −0.01)	0.020
**CDRS score**							
Pretreatment	170	1.74 (1.64–1.84)	161	1.73 (1.63–1.83)		0.003 (−0.10 to 0.10)	
Posttreatment	166	2.03 (1.74–2.32) *↑	156	1.86 (1.77–1.95) *↑	0.018	0.16 (0.06–0.026)	<0.001
**ASI-1 score (safety and security)**							
Pretreatment	170	1.52 (1.42–1.62)	161	1.53 (1.43–1.63)		−0.01 (0.09–0.08)	
Posttreatment	166	0.89 (0.82–0.96)*↓	156	1.02 (0.94–1.10)*↓	<0.001	−0.12 (−0.20 to −0.03)	<0.001
**ASI-2 score (traumatic losses and separations)**							
Pretreatment	170	1.14 (1.00–1.30)	161	1.13 (1.02–1.24)		0.002 (−0.08 to 0.08)	
Posttreatment	166	0.62 (0.55–0.69)*↓	156	0.71 (0.62–0.80)*↓	<0.001	−0.10 (−0.18 to −0.02)	0.020
**ASI-3 score (injustice)**							
Pretreatment	170	1.43 (1.27–1.59)	161	1.40 (1.24–1.56)		0.004 (−0.08 to 0.09)	
Posttreatment	166	0.95 (0.81–1.09)*↓	156	1.00 (0.87–1.13)*↓	<0.001	−0.03(−0.11 to 0.06)	0.513
**ASI-4 score (role and identity disruptions)**							
Pretreatment	170	1.28 (1.17–1.39)	161	1.30 (1.19–1.41)		−0.01 (−0.09 to 0.08)	
Posttreatment	166	0.73 (0.65–0.81)*↓	156	0.87 (0.78–0.96)*↓	<0.001	−0.12 (−0.21 to −0.04)	<0.001
**ASI-5 score (existential meaning)**							
Pretreatment	170	1.12 (1.02–1.22)	161	1.11 (1.00–1.22)		−0.03 (−0.07 to 0.07)	
Posttreatment	166	0.69 (0.62–0.76)*↓	156	0.79 (0.71–0.87)*↓	<0.001	−0.18 (−0.19 to −0.05)	<0.001
**Secondary Outcomes**							
**GAD score**							
Pretreatment	170	1.80 (1.74–1.86)	161	1.79 (1.72–1.86)		0.005 (−0.05 to 0.06)	
Posttreatment	166	1.25 (1.22–1.28)*↓	156	1.32 (1.27–1.37)*↓	<0.001	−0.08 (−0.14 to −0.02)	<0.001
**PCBD score**							
Pretreatment	170	1.66 (1.58–1.74)	105	1.55 (1.44–1.66)		0.02 (−0.05 to 0.09)	
Posttreatment	166	1.32 (1.26–1.38)*↓	101	1.42 (1.33–1.51)	0.023	−0.18 (−0.26 to −0.10)	<0.001

Note: Posttreatment was measured at 6-week follow-up.

(*↓) denotes a significant lower score from pre- to posttreatment (*p* < 0.05) and

(*↑) denotes a significant higher score from pre- to posttreatment.

^a^The adjusted analyses controlled for baseline PTSD, CPTSD, MDD, CDRS, ASI-1, ASI-2, ASI-3, ASI-4, ASI-5, GAD, and PC.

**Abbreviations:** ASI, Adaptive Stress Index; CBT, Cognitive Behavioural Therapy; CDRS, Connor–Davidson Resilience Scale; GAD, Generalized Anxiety Disorder; IAT, Integrative Adapt Therapy; MDD, Major Depressive Disorder; PCBD, Persistent Complex Bereavement Disorder; PTSD, Posttraumatic Stress Disorder

Five participants reported mild suicidal ideation (‘a little of the time’). The participants were judged as low risk because they had never attempted self-harm, had no current plan to do so, and had not obtained the means to injure themselves. Following discussion with the clinical supervisor, participants were managed following the established safety protocol with a weekly follow-up check to assess change in suicidal ideation. No participants attempted or committed suicide during the study.

### Unadjusted analyses

The unadjusted analyses indicated significant improvements in primary and secondary outcomes from baseline to 6-week posttreatment in both the IAT and the CBT arms, respectively (Table 3). The adjusted analyses controlling for baseline PTSD, CPTSD, MDD, CDRS, ASI-1, ASI-2, ASI-3, ASI-4, ASI-5, GAD, and PCBD in each model showed a consistent pattern of results in that there were significant improvements from pre- to 6-week posttreatment in the IAT and CBT arms (adjusted mean differences with 95% CIs are reported in Table 3).

### Adjusted average treatment effects

The adjusted average treatment effects (ATE) from baseline to 6-week posttreatment were all significant, indicating superiority of IAT over CBT with the exception of ASI-3. For primary outcomes, the ATE results in favour of IAT were −0.08 (95% CI: −0.14 to −0.02, *p* = 0.012) for PTSD, −0.07 (95% CI: −0.14 to −0.01, *p =* 0.025) for CPTSD, −0.07 for MDD (95% CI: −0.13 to −0.01, *p =* 0.02), 0.16 for CDRS (95% CI: 0.06–0.026, *p* ≤ 0.001), −0.12 (95% CI: −0.20 to −0.03, *p* ≤ 0.001) for ASI-1 (safety/security), −0.10 for ASI-2 (traumatic losses and separations; 95% CI: −0.18 to −0.02, *p =* 0.02), −0.03 for ASI-3 (injustice; 95% CI: −0.11 to 0.06, *p =* 0.513), −0.12 for ASI-4 (role/identity disruptions; 95% CI: −0.21 to −0.04, *p* ≤ 0.001), and −0.18 for ASI-5 (existential meaning; 95% CI: −0.19 to −0.05, *p* ≤ 0.001). For secondary outcomes, the adjusted ATE indicating superiority of IAT was −0.08 for GAD (95% CI: −0.14 to −0.02, *p* ≤ 0.001) and −0.18 for PCBD (95% CI: −0.26 to −0.10, *p* ≤ 0.001).

Effect sizes derived from model-adjusted mean differences are reported in Table 4. In comparison to CBT, with the exception of resilience (IAT, *d* = 0.20 versus CBT, *d* = 0.21), the effect sizes for primary outcomes for IAT were consistently larger for all indices including PTSD (IAT, *d* = 0.93 versus CBT, *d* = 0.87), CPTSD (*d* = 1.27 versus *d* = 1.02), MDD (*d* = 1.4 versus d = 1.11), ASI-1 (*d* = 1.1 versus *d* = 0.85), ASI-2 (*d* = 0.81 versus *d* = 0.66), ASI-3 (*d* = 0.49 versus *d* = 0.42), ASI-4 (*d* = 0.86 versus *d* = 0.67), ASI-5 (*d* = 0.72 versus *d* = 0.53); the pattern was the same for secondary outcomes: GAD *(d* = 1.67 versus *d* = 1.19), PCBD (*d* = 0.72 versus *d* = 0.25).

**Table 4:** Effect size estimates for treatment outcomes based on model-adjusted mean differences for IAT and CBT groups (*n* = 331).

Outcomes	Effect size estimate for IAT	Effect size estimate for CBT
**Primary outcomes**		
PTSD	0.93	0.87
Complex PTSD	1.27	1.02
MDD	1.40	1.11
CDRS	0.20	0.21
**ASI**		
ASI-1 score (safety and security)	1.10	0.85
ASI-2 score (traumatic losses and separations)	0.81	0.66
ASI-3 score (injustice)	0.49	0.42
ASI-4 score (role and identity disruptions)	0.86	0.67
ASI-5 score (existential meaning)	0.72	0.53
**Secondary outcomes**		
GAD	1.67	1.19
PCBD	0.72	0.25

Note: Cohen’s *d* effect sizes were calculated by dividing the difference in model-adjusted average treatment effect between treatment and control by the outcome’s pooled standard deviation at baseline. Following an established approach [43] to estimating effect size based on model-estimated mean difference, we used the baseline standard deviation of each person’s outcome in our effect size calculation as this was likely to be constant across time (over 6-week follow-up).

**Abbreviations:** ASI, Adaptive Stress Index; CBT, Cognitive Behavioural Therapy; CDRS, Connor–Davidson Resilience Scale; GAD, Generalised Anxiety Disorder; IAT, Integrative Adapt Therapy; MDD, Major Depressive Disorder; PCBD, Persistent Complex Bereavement Disorder; PTSD, Post Traumatic Stress Disorder

## Discussion

Our study is the first to test the efficacy of IAT in a direct comparison with a CBT condition. At 6-weeks posttreatment, both IAT and CBT achieved statistically significant reductions on all primary outcomes. Furthermore, IAT recorded larger ATE over CBT in reducing symptoms of all CMDs as well as in improvements in adaptive stress and resilience. Effect sizes for IAT were larger than for CBT for all primary and secondary outcomes, with the exception of resilience.

### Strengths and limitations

Strengths of the study include the systematic recruitment strategy in that participants were drawn from a representative sample of refugees from 3 community-based epidemiological studies, yielding a pool of participants that reflected the demographic profile of each; for example, our sample replicated the general pattern in the community by generating a larger sample of men amongst the Rohingya [38]. There was extensive work done to adapt and pilot the materials and procedures prior to conducting the RCT [34,35], including a systematic process of translation and adaptation of the manuals to ensure their cultural, contextual, and linguistic appropriateness. The entire intervention was conducted by lay counsellors from the respective communities, consistent with the principles of task shifting which enhances the capacity to scale up the interventions in real-life service settings. Counsellors from the 3 ethnic groups completed rigorous training and competency evaluations and demonstrated a high level of fidelity in implementing treatments under the supervision of bilingual clinical supervisors [21]. Appropriate masking was applied especially in relation to the assessment team being blinded to treatment arm allocations. Finally, there was a high retention rate (>90%) in both arms of the interventions.

Limitations of the study include the risk of cross-over—that is, the inadvertent use of techniques of one therapy when applying the other—even though supervisors made explicit this risk in training and supervision. Strategies to detect and correct this effect included regular supervision and case reviews, in vivo observations of sessions, and random checks of recorded sessions throughout implementation. We note that any cross-over effects that might have occurred would have acted to attenuate rather than accentuate differences in outcomes between the 2 therapies. An allegiance effect [45,46] must be considered, that is, the risk that the originators of IAT who initiated and oversaw the study inadvertently conveyed a preference for that modality in training and supervising counsellors. Active efforts were made to avert this bias in training and supervision, but the extent to which it influenced the results cannot be assessed. This consideration makes it imperative that independent research teams evaluate IAT in future studies. We note that issues of timeliness—the widespread interest amongst implementing agencies to use IAT—prompted us to report the initial 6-week outcomes; as indicated, the 12-month follow-up data will be reported later in 2020. We included a range of primary outcomes, which conferred the advantage of demonstrating a consistent pattern of superiority of outcomes for IAT across a range of comorbid indices. Nevertheless, we acknowledge the risk of Type I error in so doing, although that bias is unlikely to account for the overall pattern observed.

### Consideration of results in the context of the current literature

We acknowledge that the effect sizes achieved for IAT are smaller than those achieved in trials of other therapies amongst refugees. For example, the effect size for PTSD reported in a previous trial [47] comparing a transdiagnostic treatment (CETA) with a wait-list control amongst Burmese refugees in Thailand was 1.39 for moderate and 1.61 for severe PTSD; in contrast, our effect sizes for IAT were 0.93 for PTSD and 1.27 for CPTSD. The introduction of CPTSD in ICD-11 has changed the overall classification and assignment of persons to this and the PTSD category, precluding direct comparisons with past studies that preceded the application of the CPTSD diagnosis. We also note that some of the effect sizes achieved for IAT were large, for example, for MDD (1.4).

Although IAT recorded a larger treatment effect than CBT in relation to improving resilience at the 6-week follow-up, the changes for both arms were small as were the difference in effects between them. The finding may reflect the lack of specificity of the measure used in relation to the specific focus of the therapy, which, in keeping with the informing model, is focused on the construct of adaptation, or more generally, that the measure used was not wholly congruent with concepts or ways of expressing resilience in the cultures of the participating groups [18,48], noting that the transcultural universality of the concept and/or measurement of resilience remains an issue of contention [49]. In contrast, IAT achieved favourable changes on the majority of scales of the ADAPT-derived measure (noting that the difference for the ASI-3 scale of injustice was equivocal, possibly reflecting the low endorsement of items on this scale). Overall, however, the findings suggest that the ASI scales may be a more accurate measure of the changes achieved by IAT, consistent with the informing ADAPT model [28].

Finally, it is acknowledged that that the effect size differences in relation to individual outcome indices were modest in size when comparing IAT with CBT with the notable exception of some indices such as GAD and PCBD. As indicated, the generic effects of therapy limit the size of the differences that can be shown in head to head trials of this type, even when one therapy is superior to the other. Therapeutic factors common to all active therapies include the placebo effect, empathic engagement with a counsellor, cross-over of strategies used to overcome symptoms, and ceiling effects caused by those who are unresponsive to any therapy [23]—constraints recognized in both the general trauma and refugee mental health fields [50,51]. As a consequence, the ISTSS has recommended the application of a threshold of 0.25 as the effect size indicating a veridical difference when directly comparing 2 active psychotherapies in the trauma-related mental health field [42]. We note that most of our comparisons exceeded this effect size level.

### Distinctions in the conceptualisation and approach of IAT

It is important to consider the reasons for the difference in outcomes between IAT and CBT from both theoretical and clinical perspectives. From a theoretical perspective, it was anticipated that IAT would assist participants to comprehend, organise, and make sense of the major psychosocial challenges they have encountered, an understanding and awareness that would motivate them to acquire effective strategies to cope with the mental health and adaptive consequences of these changes. These strategies were applied specifically to address difficulties encountered in the domains of safety and security, traumatic losses and separations, sense of injustice, undermining of roles and identities, and loss of a sense of coherence and meaning in life. By contextualizing maladaptive personal and interpersonal responses within the individual life experience of each refugee, the techniques used to address these problems appeared to gain greater meaning and salience, motivating the person to make the necessary changes to improve their adaptive capacity and, as a consequence, their mental health.

In summary, if IAT is confirmed as an effective intervention in future studies, we suggest that there are 5 key reasons that favour the approach: (1) IAT is grounded in an explicit theoretical framework (the ADAPT model) providing a readily comprehended conceptual foundation that makes sense of the refugee experience when personalized according to the specifics of each person’s life trajectory. (2) The establishment of a coherent narrative based on the ADAPT framework helps the participant forge a meaningful account of their lives by making connections between their past and ongoing experiences and the mental health symptoms and maladaptive responses that are creating difficulties in their personal, interpersonal and social lives. (3) The insights and understandings achieved by applying IAT principles help to overcome the sense of isolation, confusion, and alienation that mental health symptoms and repetitive maladaptive interpersonal interactions evince in the person. (4) The knowledge gained motivates the participant to gain and utilize the skills provided by evidence-based techniques to address the ongoing problems identified in their personal, interpersonal, and social worlds. (5) The overall aim is not only to overcome immediate symptoms of mental distress but to build adaptive capacities that may provide the participant with a more flexible and effective way of responding to future challenges associated with their refugee status. Only longer follow-up studies will be able to demonstrate whether the final aim is achieved by IAT.

### Conclusions

The focus of existing psychotherapies for refugees tends to be on reducing symptoms of CMD, with less emphasis on linking symptoms to the specific experiences and psychosocial difficulties specific to life as a refugee. The conceptualisation and approach of IAT therefore is distinct in that, unlike conventional interventions, the therapy helps refugees trace their emotional and behavioural problems to the underlying psychosocial disruptions (reflected in the core ADAPT Pillars) they have experienced in the course of their trajectory of flight from violence and insecurity and search for a secure location of residence. This first randomized trial of IAT indicated the efficacy of the method in reducing symptoms and promoting adaptive capacity in the short-term following therapy. Further studies are needed to test whether IAT has a persisting effect in reducing mental health symptoms and adaptation amongst refugees over a prolonged period of time.

## Supporting information

S1 Text Chin kachin and rohingya refugees in malaysia. (DOCX)

S2 Text CONSORT statement. (DOCX)

S3 Text Study protocol. (DOCX)

S1 Table Competency and fidelity assessment for IAT and CBT. CBT, Cognitive Behavioural Therapy; IAT, Integrative Adapt Therapy.
(DOCX)

S2 Table Measures of mental health outcomes, adaptive stress, and resilience from the R-MHAP. R-MHAP, Refugee Mental Health Assessment Package.
(DOCX)

## Abbreviations

ADAPT: Adaptation and Development After Persecution and Trauma
ASI: Adaptive Stress Index
ATE: average treatment effects
CBT: Cognitive Behavioural Therapy
CDRS: Connor–Davidson Resilience Scale
CETA: Common Elements Treatment Approach
CMD: common mental disorders
CPTSD: Complex Post Traumatic Stress Disorder
DSM-5: Diagnostic and Statistical Manual for Mental Disorders 5^th^ Edition
ICD-11: International Classification of Diseases (11^th^ edition)
GAD: Generalised Anxiety Disorder
IAT: Integrative Adapt Therapy
IRR: Inter-Rater Reliability
MDD: Major Depressive Disorder
PCBD: Persistent Complex Bereavement Disorder
PTSD: Post Traumatic Stress Disorder
RCT: randomised controlled trial
RMHAP: Refugee Mental Health Assessment Package
