# Supplementary material for: An Integrative Adapt Therapy for common mental health symptoms and adaptive stress amongst Rohingya, Chin, and Kachin refugees living in Malaysia: A randomized controlled trial
Source: PLoS Med. 2020 Mar 31;17(3):e1003073. doi: 10.1371/journal.pmed.1003073 (PMC7108685; doi:10.1371/journal.pmed.1003073)
Supplement: S1 Text — (DOCX) [file pmed.1003073.s001.docx]

**Additional Information on Chin, Kachin, and Rohingya refugees in Malaysia**

There are currently over 150,430 refugees from Myanmar, including approximately 93,190 Rohingyas, 24,490 Chins, 9,730 Myanmar Muslims, 3,990 Rakhine & Arakanese, and other ethnicities from Myanmar. Rohingya, Chin, and Kachin although differ in their ethnicities and cultures, have all been subjected to a long history of persecution and discrimination in Myanmar. There are currently over 150,430 refugees from Myanmar living in Malaysia, including approximately 93,190 Rohingyas, 24,490 Chins, 9,730 Myanmar Muslims, 3,990 Rakhine & Arakanese, and other ethnicities from Myanmar. Rohingya, Chin, and Kachin refugees, although differ in their ethnicities and cultures, have been subjected to a long history of persecution and discrimination in Myanmar. There has been extensive documentation of widespread systematic human rights violations against ethnic minorities in Myanmar including extrajudicial killings, arbitrary arrest, detention, torture, forced labour, restrictions on movement, expression, and religious freedom, military conscription policies, extortion and confiscation of property. Many first arrived by boat in Thailand through dangerous travels over the Andaman Sea and were then smuggled or trafficked into Malaysia. They live throughout Peninsular Malaysia. All unauthorized foreigners, including persons fleeing Myanmar, are considered ‘illegal’ or ‘prohibited’ immigrants under the Immigration Act and therefore face ongoing threats of arrest and indefinite detention in deplorable conditions. For the most part, refugees in Malaysia live in overcrowded housing and under protracted conditions of statelessness with lack of access to educational opportunities, employment, and healthcare. Some refugees have lived for decades in Malaysia and have been able to set up some forms of livelihoods and/or receive remittances from relatives who managed to get resettled to Australia, Canada, Denmark, or Sweden but many continue to live in precarious economic situations. Those who have been detained in immigration detention are at risk of malnutrition, physical and mental abuse, assault, exploitation, extortion and indefinite detention.
