# Supplementary material for: An Integrative Adapt Therapy for common mental health symptoms and adaptive stress amongst Rohingya, Chin, and Kachin refugees living in Malaysia: A randomized controlled trial
Source: PLoS Med. 2020 Mar 31;17(3):e1003073. doi: 10.1371/journal.pmed.1003073 (PMC7108685; doi:10.1371/journal.pmed.1003073)
Supplement: S3 Text — (DOCX) [file pmed.1003073.s003.docx]

**A Randomized Control Trial Comparing an Integrative Adapt Therapy (IAT) with Cognitive Behavioural Treatment amongst Myanmar Refugees: A Study Protocol**

**Study synopsis**

Promoting the mental health and psychosocial wellbeing of refugees is a global public health priority. Yet questions remain about the cultural and contextual relevance and effectiveness of psychotherapies applied for specific mental health problems encountered amongst this population. First, the range of mental health problems is wide encompassing co-morbid disorders and forms of distress, yet most interventions focus on the limited outcome of post-traumatic stress disorder (PTSD). Second, western derived methods of intervention draw heavily on cognitive behavioural techniques which on their own may be culturally and contextually alien and more broadly, are not directly congruent with the experiences of refugees. Third, recruitment to studies rarely reflects a systematic approach to identifying persons in need in the community. Finally, interventions tend to be focused on psychopathology (symptoms of distress) rather than on building the capacity for positive adaptation based on an understanding of the psychosocial disruptions that refugees continue to confront following exposure to conflict and displacement. This RCT intervention is innovative in allowing all eligible refugees from a defined catchment area to participate and on focusing specifically on building adaptive capacity based on an established psychosocial model (ADAPT) amongst Burmese refugees (members of the Chin, Kachin, and Rohingya communities) in Malaysia. Specifically, the study will test the efficacy of Integrated ADAPT therapy (IAT), a novel approach that is grounded in the refugee experience rather than on a set of "imported" cognitive behavioural techniques applied to treat traumatic stress in western settings. Our team has spent the past two years in intensive engagement with the communities and relevant stakeholders, using extensive community consultation to ensure the feasibility, acceptability and safety of the project, as well as its contextual and cultural relevance. We have built a strong consensus amongst all stakeholders concerning the value and acceptability of the project. The procedure has been fully manualized, and preliminary training of potential therapists has been very well received. The comparison group will receive CBT as outlined hereunder. Integral to the study is a process for immediate translation and dissemination based on a policy and practice protocol (TPPP), a process that will be greatly facilitated by the extensive engagement, consultation and interaction process that has taken place over the preceding two years with all stakeholders relevant to the communities in which the study will be implemented

**Introduction**

It is estimated that 60 million persons have been exposed to mass conflict and displacement in the contemporary world, the largest number since WW II [1]. Questions remain about the cultural and contextual relevance and effectiveness of psychotherapies applied in the refugee mental health field [2]. Specifically, past intervention studies have been limited by (a) selection biases making it difficult to evaluate the needs of the community and the best way to engage with them and offer opportunities to obtain treatment; (b) a focus on trauma events and immediate living difficulties rather than on the longer term disruption of psychosocial support systems; (b) the use of CBT techniques derived from western models of intervention; (c) a singular focus on reducing symptoms, particularly of post-traumatic stress disorder (PTSD) and depression [3, 4]; (d) a relative under-emphasis on the explicit task of building resilience, capacity to deal with ongoing challenges, and the promotion of adaptation to the new environment; and (d) lack of follow up (most studies being limited to follow-up periods of 6 months or less); (e) failure to include an active comparison treatment and non-binding treatment providers [3, 5].

We have developed a novel psychotherapeutic approach which focuses on the stressors and adaptive difficulties caused by the undermining of the core psychosocial systems during the course of the refugee experience. Our approach, referred to as Integrative Adapt Therapy (IAT) is based on the Adaptation and Development After Persecution and Trauma (ADAPT) model [6]. The formulation, refinement, and cultural adaptation of IAT has been described previously [7].

The ADAPT model postulates that five psychosocial domains are disrupted by the sequence of events associated with the refugee experience, namely safety/security, bonds/networks, access to justice, roles/identities, and existential meaning. The model reflects a growing consensus in the field that ecosocial conditions (comprising social, cultural, economic, spiritual and existential components) shape the effects of trauma and ongoing daily living difficulties in determining the ability of individuals to adapt. Where the ecosocial environment and supportive psychosocial systems are severely disrupted, experiences of trauma and adversity are more difficult to accommodate, increasing communal and individual levels of distress and functional impairment. Importantly, the ecosocial environment itself undergoes major changes as refugees traverse the sequential phases of conflict, flight, transition and resettlement [8]. Our studies show that the disruptions of the psychosocial systems underpinning the ADAPT model play an important role in shaping mental health and adaptation outcomes and contribute not only to a wide array of comorbid mental disorders (CMDs) but also to difficulties in functioning and capacity to adapt to the new environment [9].

IAT has several advantages over conventional approaches: (a) it authenticates the refugee’s experience by tailoring the intervention to the lived experience of psychosocial disruption; (b) it is adapted to each culture and context, a process achieved by a standardized period of community consultation and research using qualitative methods (focus groups, informant interviews); (c) rather than being primarily trauma- or symptom-focused, IAT explicitly is designed to build adaptive capacity based on the individual and collective experiences of refugees, drawing on the ADAPT model; (d) the integrated process of assessment and therapy therefore aims to create a strong foundation of trust, respect and mutual understanding, thereby promoting the therapeutic alliance; (e) the integrated approach adds to the contextual validity of the intervention by focusing on key changes that have occurred during the period of conflict, flight, transition and resettlement that together have impacted on the psychosocial support systems of the community; (f) the focus is on the potential to use self-help strategies to overcoming ongoing obstacles and restoring psychosocial equilibrium insofar as possible; (g) while novel in its starting point, the therapy builds on and incorporates techniques shown to be effective in past interventions including problem-solving, behavioural activation, conflict resolution, interpersonal skills training and trauma narration, ensuring the capacity for task shifting, that is, engaging counsellors drawn from refugee groups to undertake the process [10] . Consistent with the contemporary focus and the ADAPT theory, we adopted a transdiagnostic approach in which we included a range of symptoms of CMDs frequently observed amongst refugees [11]. Specifically, we include the full range of symptoms of PTSD, Complex PTSD (CPTSD), Major Depressive Disorder (MDD), Generalized Anxiety Disorder (GAD), and Persistent Complex Bereavement Disorder (PCBD) [12, 13].

In summary, IAT aims to integrate four essential goals: to provide a clear but flexible theoretical framework that makes sense of the refugee experience; to translate these principles into the real life challenges and response patterns relevant to individuals within their culture and context; to use the understanding and insight gained as a motivator for individuals to make adaptive changes; and to provide them with the evidence-based techniques to develop adaptive strategies that are flexible enough to deal with ongoing and future challenges as they evolve.

This paper outlines the protocol for a randomized controlled trial undertaken to test the efficacy and feasibility of IAT by comparing it to an active psychological treatment, CBT^[[1]](#footnote-1)^ amongst Myanmar refugees in Malaysia. In general, refugees in Malaysia face recurring threats of arrest, detention, and deportation due to insecure residency in addition to poverty, limited educational opportunities and access to healthcare and basic services. Our study aims therefore were to test the following hypotheses: (1) that both CBT and IAT would achieve improved outcomes on all symptom measures; (2) that IAT would show a consistent pattern of superiority in outcomes compared to CBT; and (3) that IAT would demonstrate superiority compared to CBT in promoting resilience and reducing adaptive stress related to erosion of the five ADAPT pillars. The improvements will be evident immediately post-treatment and at 12-month follow-up^[[2]](#footnote-2)^.

**Study design**

In this single-blind two-armed RCT, participants who are UNHCR recognized refugees will be recruited from a clustered, multistage epidemiological study conducted amongst Myanmar refugees (from Chin, Kachin, and Rohingya communities) concentrated in and around Kuala Lumpur, the capital of Malaysia. Recruitment continued serially until we achieved the projected number of participants based on our power analysis estimates (see hereunder).

**Study participants**

Participants will comprise male and female adults (18 years of age or older) who are members of the Chin, Kachin, and Rohingya communities. Inclusion criteria are (a) meeting criteria for at least one of the included CMDs (PTSD/CPTSD, MDD, GAD, and PCBD)[11]; (b) witnessed or experienced at least one traumatic event related to mass conflict; and (c) endorsed at least one stressor on the ASI. Exclusion criteria are than 18-years of age; intellectually disabled or cognitively impaired based on obvious indicators of disability; presence of overt psychosis (as assessed using the WHO mhGAP protocol) [14].

All participants will be assessed at baseline, immediately post-treatment, and at 12-month follow-up. Figure 1 shows the flow of participants through the trial.

Figure 1. Flow Diagram

Post Intervention

Initial assessment for eligibility

Informed consent

Baseline assessment

Randomization

N=331

CBT: 6 Weekly sessions

of 45 minutes

IAT: weekly sessions

of 45 minutes

Enrolment

Allocation

6 weeks Post-treatment

assessment

Follow-up

12 months follow-up

assessment

*Sample size*

The International Society for Traumatic Stress Studies (ISTSS) has provided guidance concerning the magnitude of outcome differences that should be regarded as clinically meaningful in comparing one active psychotherapy over another in the trauma field [15]. Based on this guide, we estimated that a minimum of 150 participants were needed in each arm to achieve a moderate effect size of 0.50 and a design effect of 1.5, based on 80% power and a two-tailed 5% significance level. This calculation assumed an attrition rate of 50% in view of the pattern of substantial resettlement to third countries that could occur based on recent precedence over the period of follow-up.

*Randomization and masking*

Eligible participants will be randomized to either the IAT or CBT arm. Participants who meet the inclusion criteria will then be randomized based on a 1:1 ratio using a computer-generated randomization sequence. An off-site research assistant will oversee the randomized assignment, a process fully concealed from the counsellors who will be informed about treatment assignments via the trial coordinator. Refugees will be blind to their diagnostic status but not their treatment condition. The statistician involved in data management, analysis and interpretation of results will be blind to the participants’ treatment condition.

Following randomization, participants will attend the intervention at established NGO community centres which are trusted places of safety based on the provision within Malaysian law that affords designated NGOs legal immunity against government interference.

Field Research Personnel

*Community Intervention Teams (CIT)*

There will be three community intervention teams comprising 24 personnel, 8 personnel for each ethnic group (with an equal number of male/female counsellors within each team). All counsellors speak fluent Rohingya and Burmese and will treat participants in either the IAT or CBT arm. For cultural reasons, female counsellors will be assigned to female/male participants, whereas male counsellors will be assigned only to male participants. Each team will be trained in both IAT and CBT. To minimize cross-contamination between the treatment conditions, the trainings will be conducted separately for each ethnic group; additional care will be taken not to imply preference for one therapy over the other.

*Community Assessment Team: (CAT)* will comprise Rohingya- and Burmese speaking assessors trained (independently of CIT) specifically in administering the assessment battery at baseline, immediately post-treatment, and at 6- and 12-month follow-up. All team members will be trained in the importance of maintaining concealment, and the two teams (CIT and CAT) will be located separately.

Competency rating, supervision, and treatment fidelity

Inter-rater reliability of assessments (90% concordance in assigning cases) will be required for each assessment team member. Treatment fidelity will be assessed by independent clinical psychologists and supervisors trained in IAT or CBT based on observations of a 10% random sample of counsellor’s sessions (10 sessions per counsellor) using a checklist and narrative commentary aimed at ensuring systematic delivery of each component of the respective therapies. The supervisor will then rate each session as “satisfactory” or “unsatisfactory” based on this evaluation. Further training and fidelity checks will be provided for counsellors assigned an unsatisfactory rating. The treatment adherence evaluation has been tested in other psychotherapy. Trained counsellors from the three ethnic groups will complete rigorous competency evaluations and will be required to demonstrate a high level of fidelity in implementing treatments under the supervision of bilingual clinical supervisors.

Adaptation and delivery of treatment modules

The treatment manuals will be translated and adapted for the Rohingya, Chin, and Kachin refugees drawing on our extensive work conducted on IAT. All treatment sessions will be conducted across community offices in close proximity to the residences of the participants. Details of the process of cultural adaptation and qualitative testing have been described elsewhere [7].

*Integrative Adapt Therapy (IAT)*

As indicated, IAT is grounded in the five psychosocial pillars of the ADAPT model.[7] Refugees participating in IAT are encouraged to reflect on past and ongoing experiences that have disrupted the psychosocial foundations of their societies, their families and themselves during the trajectory of mass conflict, upheaval, displacement, flight, transition, and resettlement. They are assisted in making connections between these experiences and patterns of maladaptive personal responses that are of major concern to them in their emotional, cognitive, behavioural and interpersonal lives. By making sense of their own response patterns within the context of the disruptions experienced by themselves, their families and the refugee society as a whole, IAT sets the stage for participants to identify more precisely what changes they need to make to improve their lives. They are offered a range of skills such as problem solving and cognitive restructuring to promote their adaptive capacities in a concrete manner in order to address contemporary problems in their lives and to prepare them to navigate future challenges. As an example, refugees who experience uncontrollable episodes of aggression may be guided to draw on their experiences to make connections between their overwhelming feelings of anger and the injustices they and their families have been subjected to (Pillar III of the ADAPT model). Anger then becomes less alienating and incomprehensible returning a sense of control to the refugee who is motivated to learn strategies (cognitive, behavioural, interpersonal) that will assist in anticipating, preventing and curtailing episodes of aggression, thereby improving the sense of control and the participant’s interpersonal relationships. A similar process is followed in relation to other pillars of the ADAPT model.

*Cognitive Behavioural Treatment (CBT)*

The culturally adapted CBT condition included six core treatment strategies: psychoeducation, stress management, problem-solving, behavioural activation, cognitive reappraisal, and strengthening social support. Two criteria are applied in selecting these techniques: they include “common elements” that are evidence-based in treating symptoms of common mental disorders in populations affected by adversity; and the package is well suited to application by lay counsellors based on the principle of task-shifting [10]. Each strategy is introduced sequentially over the course of six sessions and each session built on the previously learned techniques. The emphasis is on the application of techniques to manage distressing and dysfunctional emotions and behaviours, using a range of materials and homework practice to ensure that participants mastered the skills. Although the same techniques are used in both therapies, the major difference is that the core ADAPT theme is not included in the CBT arm. Instead, the treatment is presented as an intervention to manage stress and interactions with others. Where appropriate, traumatic events are inculcated in the procedure. For example, in the session focusing on cognitive re-appraisal, a refugee who reports feelings of guilt and shame following sexual assault is taught cognitive re-appraisal techniques to address these maladaptive thoughts and feelings.

Primary outcomes

*Posttraumatic Stress Disorder, Complex PTSD, Major Depressive Disorder*

The Refugee Mental Health Assessment Package (RMHAP) [11] includes a comprehensive set of indices assessing trauma exposure, postmigration living difficulties, common mental disorders, and the Adaptive Stress Index [16], the latter recording stress arising from erosion of the five ADAPT pillars. The core modules of the RMHAP – those assessing CMDs and the ASI have been adapted and tested extensively in refugee groups [11, 16]. Revised Harvard Trauma Questionnaire (HTQ) [17] will be used to measure torture, trauma and DSM-5 PTSD symptoms.

We will use the relevant modules of the RMHAP to assess symptoms of PTSD, CPTSD, MDD, GAD and PCBD, all according to DSM-5 with the exception of CPTSD which was based on ICD-11 [11]. Symptoms are rated on a four-point scale based on how frequently they are experienced (1=not at all, 2=a little bit, 3=quite a lot, 4=extremely); the two highest frequency categories are regarded as indicating a clinically significant endorsement of the symptom. In all interviews, participants are required to complete the full symptom list for each disorder without applying skip rules. Responses were recorded on an electronic tablet thereby reducing risk of data management errors. Past psychometric analyses of both categorical and dimensional measures of the five CMDs each yielded sound internal consistency, test-retest reliability, and concurrent validity with gold-standard diagnostic measures [11, 18, 19]. A mean score was calculated for each diagnostic category and used in pre- and post-treatment outcome analyses.

*Resilience*

The Connor-Davidson Resilience Scale (CDRS) [20] consists of 25 items assessing five resiliency factors: (1) personal competence, high standards, and tenacity; (2) trust in one’s instincts, tolerance of negative affect and strengthening effects of stress; (3) positive acceptance of change, and secure relationships; (4) control; and (5) spiritual influences. The measure is the most widely used in trauma-affected populations including refugees and past studies have shown high levels of internal consistency, test-retest reliability, and convergent validity [21, 22]. Items are assessed on a 5-point Likert scale that ranges from ‘not true at all’ (scored 0) to ‘true nearly all the time’ (scored 4). Total scores range from 0 to 100; a mean score was calculated based on all items, with higher scores reflecting greater resilience.

*Adaptive Stress (ASI)*

The Adaptive Stress Index [16] comprises five individual scales, each consisting of an empirically derived set of items assessing the individual’s capacity to cope with stress arising from erosion of each ADAPT pillar: ASI-1 (12 items assessing safety and security); ASI-2 (14 items assessing traumatic losses and separations); ASI-3 (13 items assessing injustice); ASI-4 (11 items assessing role and identity disruptions); ASI-5 (14 items assessing existential meaning). Each item is scored on a 4-point Likert scale (0=not at all, 1=a little, 2=quite a lot, 3=extremely) with a possible score ranging from 0 to 3; a mean score was calculated for each ASI scale based on all scored items. The ASI as a whole as well as its five constituent scales have been subjected to extensive psychometric testing across several refugee groups [16]. Interpersonal functioning will be assessed using the Inventory of Interpersonal Problems Short Form, a brief index of individuals’ self-reported difficulties in interpersonal relationships [23].

Secondary Outcomes

Symptoms of Generalized Anxiety Disorder and Persistent Complex Bereavement Disorder will be assessed using the relevant modules of the RMHAP, as indicated above. Functionality will be assessed using the abbreviated version of the World Health Organization Disability Assessment Schedule (WHO-DAS) [24], attachment styles will be assessed using Close Relationship Scale [25] , sense of injustice will be assessed using a brief index of moral injury [26], and anticipatory traumatic stress will be assessed using a brief index drawing on our previous research in Timor-Leste [27]. A mean score was calculated for each diagnosis and used in pre and post-treatment outcome analyses.

*Adverse events reporting*

We will apply the Unwanted Events and Adverse Treatment Reaction Checklist for Psychotherapy [28] and implement procedures – developed with the community – for mitigating risk of adverse reactions using culturally adapting monitoring questions. We will ensure timely action including referral to local services if any events occur. Specifically, we will partner with UNHCR, UNHCR-affiliated health partners, Kuala Lumpur Hospital in training our team in established protocols for responding to indicators of suicidality and violence in the community.

**Analytic plan**

Statistical analysis: Planned T-tests and chi-squared tests will be applied to assess differences between the two treatment groups. Intention to treat analysis (ITT) will be applied to examine differences in baseline and post-treatment scores. All outcomes will be treated as continuous. A random-effect model will be used to estimate treatment effects including five assessment time points and counsellor ID as random effects to account for within-person correlation across time and between-correlation by counsellor. A post-hoc analysis using the baseline measurement as a covariate will be implemented. Gender, age, and other relevant sociodemographic characteristics will be examined separately as covariates. Missing data will be imputed using chain equations command in Stata which allows for pooling of data according to Rubin’s rules.

**Ethics, consent and permissions**

This study was approved by the Human Research Ethics Committee (HREC) of the University of New South Wales and Institutional Review Board, Perdana University, Malaysia. All participants will be asked to provide written consent prior to baseline and follow-up assessments and their allocated treatment.

**Discussion**

Our RCT aims to assess the efficacy and feasibility of a novel intervention, Integrative Adapt Therapy (IAT), specifically designed to strengthen the resilience and capacity of refugees to cope with the undermining of the psychosocial systems that support mental health and well-being. The overarching goal of IAT is to provide refugees with a coherent framework that assists in making sense of their experiences and their emotional and interpersonal reactions to the challenges they confront within the family and community context. As such, IAT offers an alternative approach to psychotherapeutic interventions for refugees. The adoption and wider use of IAT will await the outcome of ongoing trials, including amongst multi-ethnic Myanmar refugees, examining the efficacy, cultural, and contextual adaptability of the method. A naturalistic trial is currently being conducted amongst the Rohingya refugees in Bangladesh. Planned RCTs examining the effectiveness of IAT in primary care settings in Bangladesh and Australia will be conducted at a later stage.

References

1. UNHCR: **UNHCR projected global resettlement needs**. In: *23rd Annual Tripartite Consultations on Resettlement.* Edited by UNHCR. Geneva; 2018: 1-84.

2. Turrini G, Purgato M, Acarturk C, Anttila M, Au T, Ballette F, Bird M, Carswell K, Churchill R, Cuijpers P *et al*: **Efficacy and acceptability of psychosocial interventions in asylum seekers and refugees: systematic review and meta-analysis**. *Epidemiol Psychiatr Sci* 2019:1-13.

3. Bolton P, Lee C, Haroz EE, Murray L, Dorsey S, Robinson C, Ugueto AM, Bass J: **A transdiagnostic community-based mental health treatment for comorbid disorders: development and outcomes of a randomized controlled trial among Burmese refugees in Thailand**. *PLoS Med* 2014, **11**(11):e1001757.

4. Bass J, Murray SM, Mohammed TA, Bunn M, Gorman W, Ahmed AM, Murray L, Bolton P: **A Randomized Controlled Trial of a Trauma-Informed Support, Skills, and Psychoeducation Intervention for Survivors of Torture and Related Trauma in Kurdistan, Northern Iraq**. *Global health, science and practice* 2016, **4**(3):452-466.

5. Weiss WM, Murray LK, Zangana GA, Mahmooth Z, Kaysen D, Dorsey S, Lindgren K, Gross A, Murray SM, Bass JK *et al*: **Community-based mental health treatments for survivors of torture and militant attacks in Southern Iraq: a randomized control trial**. *BMC Psychiatry* 2015, **15**:249.

6. Silove D: **The ADAPT model: a conceptual framework for mental health and psychosocial programming in post conflict settings**. *Intervention* 2013, **11**(3):237-248.

7. Tay AK, Miah MAA, Khan S, Badrudduza M, Morgan K, Balasundaram S, Silove D: **Theoretical background, first stage development and adaptation of a novel Integrative Adapt Therapy (IAT) for refugees**. *Epidemiology and Psychiatric Sciences* 2019, **In press**.

8. Silove D: **The ADAPT model: a conceptual framework for mental health and psychosocial programming in post conflict settings** *Intervention* 2013, **11**.

9. Tay AK, Rees S, Chen J, Kareth M, Silove D: **Examining the broader psychosocial effects of mass conflict on PTSD symptoms and functional impairment amongst West Papuan refugees resettled in Papua New Guinea (PNG)**. *Soc Sci Med* 2015, **132**:70-78.

10. Murray LK, Dorsey S, Haroz E, Lee C, Alsiary MM, Haydary A, Weiss WM, Bolton P: **A common elements treatment approach for adult mental health problems in low-and middle-income countries**. *Cognitive and behavioral practice* 2014, **21**(2):111-123.

11. Tay AK, Rees S, Chen J, Kareth M, Mohsin M, Silove D: **The Refugee-Mental Health Assessment Package (R-MHAP); rationale, development and first-stage testing amongst West Papuan refugees**. *International journal of mental health systems* 2015, **9**(1):1-13.

12. Tay AK, Rees S, Tam N, Kareth M, Silove D: **Defining a combined constellation of complicated bereavement and PTSD and the psychosocial correlates associated with the pattern amongst refugees from West Papua**. *Psychological medicine* 2018:1-9.

13. Tay AK, Rees S, Liddell B, Tam N, Nickerson A, Steel C, Silove D: **The role of grief symptoms and a sense of injustice in the pathways to post-traumatic stress symptoms in post-conflict Timor-Leste**. *Epidemiology and Psychiatric Sciences* 2016, **In press**.

14. World Health Organization: **mhGAP Humanitarian Intervention Guide ( mhGAP-HIG) for mental, neurological and substance use conditions in humanitarian emergencies: mental health global Action Programme (‎‎‎mhGAP)‎‎‎**: World Health Organization; 2015.

15. ISTSS: **Posttraumatic Stress Disorder Prevention and Treatment Guidelines: Methodology and Recommendations**. In*.* USA: Chicago International Society for Traumatic Stress Studies; 2019.

16. Tay AK, Rees S, Tam N, Kareth M, Silove D: **Developing a measure of adaptive stress arising from the psychosocial disruptions experienced by refugees based on a sample of displaced persons from West Papua**. *International Journal of Methods in Psychiatric Research* 2019, **1770**:1-11.

17. Berthold SM, Mollica RF, Silove D, Tay AK, Lavelle J, Lindert J: **The HTQ-5: revision of the Harvard Trauma Questionnaire for measuring torture, trauma and DSM-5 PTSD symptoms in refugee populations**. *European journal of public health* 2018, **29**(3):468-474.

18. Tay AK, Mohsin M, Rees S, Tam N, Kareth M, Silove D: **Factor structures of Complex Posttraumatic Stress Disorder and PTSD in a community sample of refugees from West Papua**. *Comprehensive psychiatry* 2018, **85**:15-22.

19. Tay AK, Rees S, Chen J, Kareth M, Silove D: **Factorial structure of complicated grief: associations with loss-related traumatic events and psychosocial impacts of mass conflict amongst West Papuan refugees**. *Soc Psychiatry Psychiatr Epidemiol* 2015.

20. Connor KM, Davidson JR: **Development of a new resilience scale: the Connor-Davidson Resilience Scale (CD-RISC)**. *Depress Anxiety* 2003, **18**(2):76-82.

21. Ahmed AS: **Post-traumatic stress disorder, resilience and vulnerability**. *Advances in Psychiatric Treatment* 2007, **13**:369--375.

22. Ziaian T, de Anstiss H, Antoniou G, Baghurst P, Sawyer M: **Resilience and Its Association with Depression, Emotional and Behavioural Problems, and Mental Health Service Utilisation among Refugee Adolescents Living in South Australia**. *International Journal of Population Research* 2012, **1-9**.

23. Barkham M, Hardy GE, Startup M: **The IIP‐32: A short version of the Inventory of Interpersonal Problems**. *British Journal of Clinical Psychology* 1996, **35**(1):21-35.

24. Üstün TB, Chatterji S, Kostanjsek N, Rehm J, Kennedy C, Epping-Jordan J, Saxena S, Korff Mv, Pull C: **Developing the World Health Organization disability assessment schedule 2.0**. *Bulletin of the World Health Organization* 2010, **88**:815-823.

25. Wei M, Russell DW, Mallinckrodt B, Vogel DL: **The Experiences in Close Relationship Scale (ECR)-short form: Reliability, validity, and factor structure**. *Journal of personality assessment* 2007, **88**(2):187-204.

26. Nash WP, Marino Carper TL, Mills MA, Au T, Goldsmith A, Litz BT: **Psychometric evaluation of the moral injury events scale**. *Military Medicine* 2013, **178**(6):646-652.

27. Tay AK, Rees S, Chen J, Kareth M, Silove D: **Pathways involving traumatic losses, worry about family, adult separation anxiety and posttraumatic stress symptoms amongst refugees from West Papua**. *Journal of anxiety disorders* 2015, **35**:1-8.

28. Linden M: **How to define, find and classify side effects in psychotherapy: from unwanted events to adverse treatment reactions**. *Clin Psychol Psychother* 2013, **20**(4):286-296.

1. We note that the CBT control was drawn from the elements of PM+ (see Table 1), on reconsideration, we determined to remove the designation of the control group as “PM+” even though the intervention we used is consistent with that approach. We made the change because a) our counsellors did not receive formal training from a certified PM+ trainer; b) the treatment components are consistent with a broad range of CBT-based approaches and this should be emphasized, as detailed in Table 1; c) our CBT arm involves 6 sessions whereas PM+ involves five session – the extension made ensure comparability in duration and timing of the therapy with the IAT arm. [↑](#footnote-ref-1)
2. We recognized during the course of the study that the initial design to undertake further assessments at 3 months, 6 months and 12 months would not be feasible for two reasons: firstly, it would overburden participants who are under substantial duress coping with everyday life challenges and work; and second, because of the logistic issues of tracing persons repeatedly as they move from one often temporary job to another. For that reason, we altered the design to follow-up at 6 weeks and 12 months. [↑](#footnote-ref-2)
