# Supplementary material for: An Integrative Adapt Therapy for common mental health symptoms and adaptive stress amongst Rohingya, Chin, and Kachin refugees living in Malaysia: A randomized controlled trial
Source: PLoS Med. 2020 Mar 31;17(3):e1003073. doi: 10.1371/journal.pmed.1003073 (PMC7108685; doi:10.1371/journal.pmed.1003073)
Supplement: S1 Table — CBT, Cognitive Behavioural Therapy; IAT, Integrative Adapt Therapy. (DOCX) [file pmed.1003073.s004.docx]

**IAT: Clinical Skills Assessment Mark Sheet**

In the Stage-II Clinical Skills Assessment, each IAT trainee provider will need to complete minimum 10 sessions over the period of 4-week on-the-job training. Trainees’ clinical skills and competencies will be rated by two independent supervisors who will observe their sessions with clients. *To proceed to Stage III, the trainee will need to score at least a “2” (pass) for 80% of the items in both the general counselling skills and IAT-specific clinical skills components.*

IAT trainee provider name: __________________________

Session: __________________________

***Supervisor: assign a score (1 = poor/fail, 2 = pass, 3 = excellent, n/a = non-applicable) to each skill-based component.***

| **General counselling skills** | **Score** | **Benchmark indicators** |
| --- | --- | --- |
| Establish rapport |  | *Did the trainee greet and introduce appropriately?* |
| Listen attentively |  | *Did the trainee listen while the client is talking?* |
| Paraphrasing |  | *Did the trainee paraphrase and break down complex concepts to make sure the client understands?* |
| Use of open-ended questions |  | *Did the trainee use a mixture of open and close-ended questions?* |
| Use of emphatic statements |  | *Did the trainee use emphatic statements (e.g. I am sorry to hear that. That must be really hard for you)?* |
| Explain confidentiality issues |  | *Did the trainee explain confidentiality issues?* |
| Culturally sensitive |  | *Did the trainee show culturally sensitivity in his/her dealing with the client?* |
| Engaging/motivating client |  | Did the trainee engage and motivate the client when he/she shows reluctance in participating? |
| Appropriate use of language |  | *Did the trainee show a good grasp of the local language (e.g. concepts, terminology, idioms) to a sufficient degree to communicate with the client?* |
| **IAT-G specific group skills (Applicable/Non-Applicable)** | **Score** | **Benchmark indicators** |
| Group leadership skills |  | *Did the trainee shows group leadership skills in IAT-G?* |
| Setting group rules |  | *Did the trainee set group rules in the first session?* |
| Encourage group participation |  | *Did the trainee encourage group members to participate?* |
| Manage group conflict |  | *Did the trainee show creative, culturally appropriate, sensitive ways of managing group conflict?* |
| Understand group dynamics |  | *Did the trainee show understanding of group dynamics and use it in appropriate ways to maintain group cohesion and participation?* |
| Notes: | | |

***Supervisor: assign a score (1 = poor/fail, 2 = pass, 3 = excellent, n/a = non-applicable) to each skill-based component.***

| **IAT Strategies/components** | **Sub-components** | **Global Clinical Skills Appraisal Criteria and Scoring Guide** | **Score** |
| --- | --- | --- | --- |
| Psychoeducation | 1. Greet and introduce 2. Explain the ADAPT model 3. Explain refugee mental health problems 4. Explain IAT 5. Motivate participation 6. Confidentiality | *1=poor/fail. The trainee failed to deliver this component in the session.*  *2=pass. The trainee delivered some but not all of the sub-components in the session.*  *3=excellent. The trainee delivered ALL of the sub-components of psychoeducation in the session.* |  |
| Notes | | | |
| Story telling | 1. Identity significant events 2. Narrate chronologically 3. Contextualize the client’s experience according to the relevant ADAPT Pillars 4. Help the client narrate a chronological history of events from their perspectives. | *1=poor/fail. The trainee failed to deliver this component in the session. The trainee was not able to elicit a relevant history from the client; or focused excessively on a particular event.*  *2=pass. The trainee was able to elicit a history and covered the major events in the client’s life according to the ADAPT domains.*  *3=excellent. The trainee was able to use the appropriate tools to elicit a history chronologically related to the ADAPT domains and allowed the client to tell their stories from their perspectives.* |  |
| Notes: | | | |
| Problem-solving | 1. Identity at least 3 problems arising from the relevant ADAPT Pillars 2. Exploring problems/symptoms underlying these problems 3. Explore coping strategies 4. Explore barriers 5. Explore solutions | *1=poor/fail. The trainee failed to deliver this part of the session.*  *2=pass. The trainee delivered some but not all of the sub-components of problem-solving.*  *3=excellent. The trainee delivered ALL of the sub-components of problem-solving in this session.* |  |
| Notes: | | | |
| Stress management | 1. Introduce the concept of stress management 2. Teach steps of controlled breathing using a prepared culturally adapted script 3. Teach steps of progressive muscle relaxation using a prepared script 4. Repeat and practice the steps | *1=poor/fail. The trainee failed to deliver this component in the session.*  *2=pass. The trainee delivered this component fairly well.*  *3=excellent. The trainee delivered this component in line with professional standards.* |  |
| Notes: | | | |
| Emotion regulation | 1. Introduce the concept of emotion regulation in refugee mental health 2. Identity/label emotions related to the problems encountered following the ADAPT Pillars 3. Normalize the client’s distress 4. Teach the client skills to develop self-awareness of these emotions, accept and address these reactions without placing judgement on them; making a distance between self and these emotions in a rational manner | *1=poor/fail. The trainee failed to deliver this component in the session.*  *2=pass. The trainee delivered some but not all of the sub-components of emotion regulation.*  *3=excellent. The trainee delivered ALL of the sub-components of emotion regulation* |  |
| Notes: | | | |
| Cognitive reappraisal | 1. Introduce the concept of cognitive reappraisal 2. Identify negative problematic thinking 3. Explore alternative ways of thinking 4. Challenge the client’s thinking in view of the reality vs expectations | *1=poor/fail. The trainee failed to deliver this component in the session.*  *2=pass. The trainee was able to help the client explore different ways of thinking.*  *3=excellent. The trainee was able to listen and challenge the client’s problematic thoughts within reasons and highlight alternative ways of thinking. In addition, the trainee helped the client recognize and address the conflict between expectations and the reality.* |  |
| Strengthen social support | 1. Introduce the client to the link between social isolation and mental health problems 2. Identify trusted friends and family members 3. Teach communication skills to address interpersonal conflict | *1=poor/fail. The trainee failed to deliver this component in the session.*  *2=pass. The trainee helped the client connect with trusted friends and family members.*  *3=excellent. The trainee did a superb job in connecting the client with trusted friends and family members and showing the client how to manage interpersonal conflict.* |  |
| Notes: | | | |
| Meaning making | 1. Introduce the concept of meaning making 2. Help the client make sense of the reality and accept the reality without judgement 3. Help the client explore the possibility of living a valued life 4. Give hope 5. Help the client find a sense of gratitude meaning in life 6. Commit to goals | *1=poor/fail. The trainee failed to deliver this component in the session.*  *2=pass. The trainee delivered some but not all of the sub-components in the session.*  *3=excellent. The trainee delivered ALL sub-components in the session.* |  |
| Notes: | | | |

**CBT: Clinical Skills Assessment Mark Sheet**

In the Stage-II Clinical Skills Assessment, each IAT trainee provider will need to complete minimum 10 sessions over the period of 4-week on-the-job training. Trainees’ clinical skills and competencies will be rated by two independent supervisors who will observe their sessions with clients. *To proceed to Stage III, the trainee will need to score at least a “2” (pass) for 80% of the items in both the general counselling skills and IAT-specific clinical skills components.*

*N.B: The CBT condition involves six weekly 45-minute sessions and included six core treatment strategies: psychoeducation, stress management, problem-solving, behavioural activation, cognitive reappraisal, and strengthening social support. We note that these elements (except for cognitive reappraisal), which have been adapted and tested in several groups, are now included in the Problem Management Plus manual developed by the World Health Organization. The six CBT sessions outlined hereunder are drawn from the PM+ manual and adapted for this trial.*

IAT trainee provider name: __________________________

Session: __________________________

***Supervisor: assign a score (1 = poor/fail, 2 = pass, 3 = excellent, n/a = non-applicable) to each skill-based component.***

| **General counselling skills** | **Score** | **Benchmark indicators** |
| --- | --- | --- |
| Establish rapport |  | *Did the trainee greet and introduce appropriately?* |
| Listen attentively |  | *Did the trainee listen while the client is talking?* |
| Paraphrasing |  | *Did the trainee paraphrase and break down complex concepts to make sure the client understands?* |
| Use of open-ended questions |  | *Did the trainee use a mixture of open and close-ended questions?* |
| Use of emphatic statements |  | *Did the trainee use emphatic statements (e.g. I am sorry to hear that. That must be really hard for you)?* |
| Explain confidentiality issues |  | *Did the trainee explain confidentiality issues?* |
| Culturally sensitive |  | *Did the trainee show culturally sensitivity in his/her dealing with the client?* |
| Engaging/motivating client |  | Did the trainee engage and motivate the client when he/she shows reluctance in participating? |
| Appropriate use of language |  | *Did the trainee show a good grasp of the local language (e.g. concepts, terminology, idioms) to a sufficient degree to communicate with the client?* |
| **CBT specific group skills (Applicable/Non-Applicable)** | **Score** | **Benchmark indicators** |
| Group leadership skills |  | *Did the trainee shows group leadership skills in IAT-G?* |
| Setting group rules |  | *Did the trainee set group rules in the first session?* |
| Encourage group participation |  | *Did the trainee encourage group members to participate?* |
| Manage group conflict |  | *Did the trainee show creative, culturally appropriate, sensitive ways of managing group conflict?* |
| Understand group dynamics |  | *Did the trainee show understanding of group dynamics and use it in appropriate ways to maintain group cohesion and participation?* |
| Notes: | | |

***Supervisor: assign a score (1 = poor/fail, 2 = pass, 3 = excellent, n/a = non-applicable) to each session (and strategies delivered within each session).***

| **CBT sessions** | **Strategies assessed** | **Global Clinical Skills Appraisal Criteria** | **Score** |
| --- | --- | --- | --- |
| Session 1: Psychoeducation | 1. Introduction and confidentiality 2. What is CBT 3. Understanding how adversity impacts on mental health 4. Managing stress 5. Ending session | *1=poor/fail. The trainee failed to deliver this session.*  *2=pass. The trainee delivered some but not all of the sub techniques in the session.*  *3=excellent. The trainee delivered ALL of the techniques in this session.* |  |
| Session 2: Problem-solving, managing stress | 1. General review 2. Managing problems 3. Managing stress 4. Ending session | *1=poor/fail. The trainee failed to deliver this session.*  *2=pass. The trainee delivered some but not all of the sub techniques in the session.*  *3=excellent. The trainee delivered ALL of the techniques in this session.* |  |
| Session 3: Problem-solving, managing stress, and behavioural activation | 1. General review 2. Managing problems 3. Get going, keep going 4. Managing stress 5. Ending the session | *1=poor/fail. The trainee failed to deliver this session.*  *2=pass. The trainee delivered some but not all of the sub techniques in the session.*  *3=excellent. The trainee delivered ALL of the techniques in this session.* |  |
| Session 4: Problem-solving, managing stress, and behavioural activation, strengthening social support | 1. General review 2. Managing problems 3. Get going, keep going 4. Strengthening social support 5. Managing stress 6. Ending the session | *1=poor/fail. The trainee failed to deliver this session.*  *2=pass. The trainee delivered some but not all of the sub techniques in the session.*  *3=excellent. The trainee delivered ALL of the techniques in this session.* |  |
| Session 5: Problem-solving, managing stress, and behavioural activation, strengthening social support, cognitive reappraisal | 1. General review 2. Managing problems 3. Get going, keep going 4. Strengthening social support 5. Managing stress 6. Cognitive reappraisal 7. Ending the session | *1=poor/fail. The trainee failed to deliver this session.*  *2=pass. The trainee delivered some but not all of the sub techniques in the session.*  *3=excellent. The trainee delivered ALL of the techniques in this session.* |  |
| 6. Ending treatment | 1. General review 2. Staying Well 3. Imagining how to help others 4. Looking to the future 5. Ending the program | *1=poor/fail. The trainee failed to deliver this session.*  *2=pass. The trainee delivered some but not all of the sub techniques in the session.*  *3=excellent. The trainee delivered ALL of the techniques in this session.* |  |
| Notes: | | | |
