# Supplementary material for: An Integrative Adapt Therapy for common mental health symptoms and adaptive stress amongst Rohingya, Chin, and Kachin refugees living in Malaysia: A randomized controlled trial
Source: PLoS Med. 2020 Mar 31;17(3):e1003073. doi: 10.1371/journal.pmed.1003073 (PMC7108685; doi:10.1371/journal.pmed.1003073)
Supplement: S2 Table — R-MHAP, Refugee Mental Health Assessment Package. (DOCX) [file pmed.1003073.s005.docx]

**Text S1** Measures of mental health outcomes, adaptive stress, and resilience from the Refugee Mental Health Assessment Package (R-MHAP).

| ***INTERVIEWER: Sometimes people experience terrifying things. They get really upset talking about these things as they bring back bad memories and feelings. I’d like to ask you some questions about the most terrifying thing(s) that you have experienced. You can tell me as much or little as you want; I am interested in how this might affect you. The information your provide us will help us to find the best ways to help you in the future. Before I start I want to make sure that you are OK. Can we start? Can you remember the most terrifying thing(s) that happened to you?*** | | | | | | | | | | |
| --- | --- | --- | --- | --- | --- | --- | --- | --- | --- | --- |
| POSTTRAUMATIC STRESS DISORDER | | | | | | | | | | |
| **Have you directly experienced or witnessed events involving:** | | | **YES** | | | **NO** | |  | |  |
| 1. Death (Specify when) | | | **YES** | | | **NO** | |  | |  |
| 1. Serious injury (Specify when) | | | **YES** | | | **NO** | |  | |  |
| 1. Sexual violation (Specify when) | | | **YES** | | | **NO** | |  | |  |
| 1. Others (Specify when): | | | **YES** | | | **NO** | |  | |  |
| **INTERVIEWER: Now I want you to focus on the event(s) you find most upsetting and terrifying. I’ll ask you some questions about some of the symptoms that you have after that event.** | | | | | | | | | | |
| 1. Have you experienced this yourself? | | | **YES** | | | **NO** | |  | |  |
| 1. Have you witnessed in person the event(s) as they happened to others? | | | **YES** | | | **NO** | |  | |  |
| **Following the event, during the LAST 4 WEEKS, have you…** | | | | | | | | | | |
| 1. Suddenly thought about the event(s) though you did not want to think about it? | **NOT AT ALL** | | | **A LITTLE** | | | **QUITE A LOT** | | **EXTREMELY** | |
| 1. Had nightmares about the event(s)? | **NOT AT ALL** | | | **A LITTLE** | | | **QUITE A LOT** | | **EXTREMELY** | |
| 1. Felt or behaved as though the event(s) were happening again when you were awake? (Note: with the extreme expression being losing touch of present surroundings)? | **NOT AT ALL** | | | **A LITTLE** | | | **QUITE A LOT** | | **EXTREMELY** | |
| 1. Got very upset when reminded of the event(s)? | **NOT AT ALL** | | | **A LITTLE** | | | **QUITE A LOT** | | **EXTREMELY** | |
| 1. had strong physical reactions (e.g., feeling dizzy, heart racing, sweating, shaking, short of breath, chest pain, stomach pain) when reminded of the event(s)? | **NOT AT ALL** | | | **A LITTLE** | | | **QUITE A LOT** | | **EXTREMELY** | |
| 1. Tried to avoid thinking about the event? | **NOT AT ALL** | | | **A LITTLE** | | | **QUITE A LOT** | | **EXTREMELY** | |
| 1. Tried to avoid people, places, talking, activities, things, and situations about the event? | **NOT AT ALL** | | | **A LITTLE** | | | **QUITE A LOT** | | **EXTREMELY** | |
| 1. Had difficulty remembering some important parts of the event (not due to head injury, alcohol, drugs, or amnesia)? | **NOT AT ALL** | | | **A LITTLE** | | | **QUITE A LOT** | | **EXTREMELY** | |
| 1. Had strong negative beliefs about yourself, others, or the world? (e.g. that you are a bad person, you can trust no one, you’ve lost your soul, or the world is very dangerous place)? | **NOT AT ALL** | | | **A LITTLE** | | | **QUITE A LOT** | | **EXTREMELY** | |
| 1. Blamed yourself or others constantly for the event or what happened as a result of the event(s)? | **NOT AT ALL** | | | **A LITTLE** | | | **QUITE A LOT** | | **EXTREMELY** | |
| 1. Had strong negative feelings (e.g. shame, fear, horror, anger, guilt)? | **NOT AT ALL** | | | **A LITTLE** | | | **QUITE A LOT** | | **EXTREMELY** | |
| 1. Lost interest in things that you used to enjoy (e.g. reading, walking, socializing, etc)? | **NOT AT ALL** | | | **A LITTLE** | | | **QUITE A LOT** | | **EXTREMELY** | |
| 1. Felt cut off or tried to stay away from people? | **NOT AT ALL** | | | **A LITTLE** | | | **QUITE A LOT** | | **EXTREMELY** | |
| 1. Had difficulty experiencing positive emotions (e.g., love happiness, joy)? | **NOT AT ALL** | | | **A LITTLE** | | | **QUITE A LOT** | | **EXTREMELY** | |
| 1. Felt hopeless about the future? | **NOT AT ALL** | | | **A LITTLE** | | | **QUITE A LOT** | | **EXTREMELY** | |
| 1. Felt irritable, angry, or aggressive* towards people? | **NOT AT ALL** | | | **A LITTLE** | | | **QUITE A LOT** | | **EXTREMELY** | |
| 1. Tried to do something that you know may cause you or other people harm? | **NOT AT ALL** | | | **A LITTLE** | | | **QUITE A LOT** | | **EXTREMELY** | |
| 1. Felt suddenly scared for no reason? | **NOT AT ALL** | | | **A LITTLE** | | | **QUITE A LOT** | | **EXTREMELY** | |
| 1. Been on guard constantly even when there was no real need to be? | **NOT AT ALL** | | | **A LITTLE** | | | **QUITE A LOT** | | **EXTREMELY** | |
| 1. Had difficulty concentrating (e.g. at work, school, etc.)? | **NOT AT ALL** | | | **A LITTLE** | | | **QUITE A LOT** | | **EXTREMELY** | |
| 1. Had trouble falling or staying asleep? | **NOT AT ALL** | | | **A LITTLE** | | | **QUITE A LOT** | | **EXTREMELY** | |
| 1. Have these symptoms lasted for ONE MONTH or more? | | **YES** | | | **NO** | |  | |  | |
| 1. Onset: When did you FIRST start having these symptoms [how many months ago]? | | Record:__________ | | | | | | | | |
| 1. Have you had these symptoms for more than 3 months | | **YES** | | | **NO** | |  | |  | |

| **INTERVIEWER: *Sometimes we feel upset because of the problems we are having in our lives and what happened in the past. I’d like to ask you questions about some of these symptoms that may affect you.*** | | | | | | | |
| --- | --- | --- | --- | --- | --- | --- | --- |
| MAJOR DEPRESSION | | | | | | | |
| **During the 2-week period in the PAST MONTH, have you…** | | | | | | | |
| 1. Felt sad or down even if someone tries to cheer you up? | **NOT AT ALL** | **A LITTLE** | | **QUITE A LOT** | | | **EXTREMELY** |
| 1. Lost interest in things (e.g., gardening, reading, etc.)? | **NOT AT ALL** | **A LITTLE** | | **QUITE A LOT** | | | **EXTREMELY** |
| 1. Lost or increased appetite? | **NOT AT ALL** | **A LITTLE** | | **QUITE A LOT** | | | **EXTREMELY** |
| 1. Had trouble falling or staying asleep? | **NOT AT ALL** | **A LITTLE** | | **QUITE A LOT** | | | **EXTREMELY** |
| 1. Felt restless or couldn’t sit still? | **NOT AT ALL** | **A LITTLE** | | **QUITE A LOT** | | | **EXTREMELY** |
| 1. Felt low in energy or tired for no reason? | **NOT AT ALL** | **A LITTLE** | | **QUITE A LOT** | | | **EXTREMELY** |
| 1. Blamed yourself for things? | **NOT AT ALL** | **A LITTLE** | | **QUITE A LOT** | | | **EXTREMELY** |
| 1. Felt worthless? | **NOT AT ALL** | **A LITTLE** | | **QUITE A LOT** | | | **EXTREMELY** |
| 1. Had trouble concentrating or difficulty making decision? | **NOT AT ALL** | **A LITTLE** | | **QUITE A LOT** | | | **EXTREMELY** |
| 1. Thought of ending your life? | **NOT AT ALL** | **A LITTLE** | | **QUITE A LOT** | | | **EXTREMELY** |
| 1. Have these symptoms lasted during the same two-week period in the LAST MONTH? | **NOT AT ALL** | **A LITTLE** | | **QUITE A LOT** | | | **EXTREMELY** |
| 1. Onset: When did you start having these symptoms [how many months ago]? | **RECORD:___________** | | | | | | |
| 1. **If onset more** than 2 years (24 months) > chronic /dysthymic | **YES** | | | | | **NO** | |
| 1. Overall, have these symptoms significantly affected your life and ability to do things you normally do in the past TWO WEEKS? | **NOT AT ALL** | | **A LITTLE** | | **QUITE A LOT** | | **EXTREMELY** |

| **INTERVIEWER: *Sometimes we worry a lot about things. These worries can affect our health. Now I’d like to ask you some questions about what worries you and how that may affect you.*** | | | | | | | | | | | | |  |  |
| --- | --- | --- | --- | --- | --- | --- | --- | --- | --- | --- | --- | --- | --- | --- |
| GENERALIZED ANXIETY DISORDER | | | | | | | | | | | | |  |  |
| **In the last WEEK, have you…** | | | | | | | | | | | | |  |  |
| worried and felt anxious a lot about things such as : | | | | | | | | | | | | |  |  |
| 1. Housing? | **NOT AT ALL** | | **A LITTLE** | | | | **QUITE A LOT** | | | | **EXTREMELY** | |  |  |
| 1. Children not going to school? | **NOT AT ALL** | | **A LITTLE** | | | | **QUITE A LOT** | | | | **EXTREMELY** | |  |  |
| 1. Income or financial status? | **NOT AT ALL** | | **A LITTLE** | | | | **QUITE A LOT** | | | | **EXTREMELY** | |  |  |
| 1. Family? (e.g., conflict in family) | **NOT AT ALL** | | **A LITTLE** | | | | **QUITE A LOT** | | | | **EXTREMELY** | |  |  |
| 1. Physical or mental health? | **NOT AT ALL** | | **A LITTLE** | | | | **QUITE A LOT** | | | | **EXTREMELY** | |  |  |
| 1. Loss of family members? | **NOT AT ALL** | | **A LITTLE** | | | | **QUITE A LOT** | | | | **EXTREMELY** | |  |  |
| 1. Others (Specify): | **NOT AT ALL** | | **A LITTLE** | | | | **QUITE A LOT** | | | | **EXTREMELY** | |  |  |
| 1. Have you had difficulty controlling the worry? | **NOT AT ALL** | | **A LITTLE** | | | | **QUITE A LOT** | | | | **EXTREMELY** | |  |  |
| 1. Have the worries occurred more than 3 months, almost every day? | **YES** | | **NO** | | | |  | | | |  | |  |  |
| 1. Have the worries occurred more than 6 months, almost every day? | **YES** | | **NO** | | | |  | | | |  | |  |  |
| 1. Onset: When did you start having these symptoms [how many months ago]? |  | | | | | | | | | | | |  |  |
| **Because of the worry and anxiety, have you…** | | | | | | | | | | | | |  |  |
| 1. Felt restless, or couldn’t sit still? | **NOT AT ALL** | | **A LITTLE** | | | | **QUITE A LOT** | | | | **EXTREMELY** | |  |  |
| 1. Felt tense or on edge? | **NOT AT ALL** | | **A LITTLE** | | | | **QUITE A LOT** | | | | **EXTREMELY** | |  |  |
| 1. Felt tired easily? | **NOT AT ALL** | | **A LITTLE** | | | | **QUITE A LOT** | | | | **EXTREMELY** | |  |  |
| 1. Had trouble concentrating because worrying too much? | **NOT AT ALL** | | **A LITTLE** | | | | **QUITE A LOT** | | | | **EXTREMELY** | |  |  |
| 1. Had trouble sleeping because worrying too much? | **NOT AT ALL** | | **A LITTLE** | | | | **QUITE A LOT** | | | | **EXTREMELY** | |  |  |
| 1. Felt irritable because worrying too much? | **NOT AT ALL** | | **A LITTLE** | | | | **QUITE A LOT** | | | | **EXTREMELY** | |  |  |
| 1. Tried to avoid things that you worry about? | **NOT AT ALL** | | **A LITTLE** | | | | **QUITE A LOT** | | | | **EXTREMELY** | |  |  |
| 1. Put things off because you worry? | **NOT AT ALL** | | **A LITTLE** | | | | **QUITE A LOT** | | | | **EXTREMELY** | |  |  |
| 1. Spent a lot of time and effort preparing for things because you worry? | **NOT AT ALL** | | **A LITTLE** | | | | **QUITE A LOT** | | | | **EXTREMELY** | |  |  |
| 1. Tried to do other things to avoid worrying too much? | **NOT AT ALL** | | **A LITTLE** | | | | **QUITE A LOT** | | | | **EXTREMELY** | |  |  |
| 1. Sought help from people or things (e.g. alcohol or other things) to calm down your worries? | **NOT AT ALL** | | **A LITTLE** | | | | **QUITE A LOT** | | | | **EXTREMELY** | |  |  |
| 1. Overall, have these symptoms significantly affected your life and ability to do things you normally do in the past month? | **NOT AT ALL** | | **A LITTLE** | | | | **QUITE A LOT** | | | | **EXTREMELY** | | |  |
|  | | | | | | | | | | | | |  |  |
|  | | | | | | | | | | | | |  |  |
|  | | | | | | | | | | | | | | |
| INTERVIEWER: ***Sometimes we feel very upset after people we loved died.***  ***Thinking too much about this can affect our health in the long-term. Now I’d like to ask you some questions about the person whose death has left you feeling very upset.*** | | | | | | | | | | | | | | |
| PERSISTENT COMPLEX BEREAVEMENT-RELATED DISORDER | | | | | | | | | | | | | | |
| 1. Have you experienced the death of someone (e.g., a family member, close friend) 12 months ago or more? | **YES** | | **NO** | | | | |  |  |  |  |  |  |  |
| 1. **[IF YES] go**to PCBD-A3> specify who:____________________________ 2. **IF no, go to next module (SAD)** | | | | | | | | | | | | | | |
| 1. If more than 1 death identified > can you tell me one of the people where the death has left you very upset for more than 12 months?   Specify how did the person die:   1. Sickness 2. Accidents (e.g. drowning, fell down from construction building during work) 3. Murders 4. Animal attacks (e.g. tiger, elephant, snake) 5. Natural disasters 6. Other ____________________ | | | | | | | | | | | | | | |
| **Since the person’s death, have you….** | | | | | | | | | | | | | | |
| 1. Had strong feelings (yearnings/longings) for the person is dead? | **NOT AT ALL** | | | **A LITTLE** | | | | **QUITE A LOT** | | | | **EXTREMELY** | | |
| 1. Felt very sorrow and painful about the death? | **NOT AT ALL** | | | | **A LITTLE** | | | **QUITE A LOT** | | | | **EXTREMELY** | | |
| 1. Thought a lot about the person who is dead? | **NOT AT ALL** | | | | **A LITTLE** | | | **QUITE A LOT** | | | | **EXTREMELY** | | |
| 1. Thought a lot about how or why the person died? | **NOT AT ALL** | | | | **A LITTLE** | | | **QUITE A LOT** | | | | **EXTREMELY** | | |
| 1. Experienced these symptoms nearly every day? | **NOT AT ALL** | | | | **A LITTLE** | | | **QUITE A LOT** | | | | **EXTREMELY** | | |
| 1. Been bothered very much by these symptoms? | **NOT AT ALL** | | | | **A LITTLE** | | | **QUITE A LOT** | | | | **EXTREMELY** | | |
| 1. Had great trouble accepting that the person is gone? | **NOT AT ALL** | | **A LITTLE** | | | | | | **QUITE A LOT** | | | **EXTREMELY** | | |
| 1. Felt shocked, stunned, or unable to feel anything? | **NOT AT ALL** | | **A LITTLE** | | | | | | **QUITE A LOT** | | | **EXTREMELY** | | |
| 1. Had difficulty remembering good things about or happy times spent with the person? | **NOT AT ALL** | | **A LITTLE** | | | | | | **QUITE A LOT** | | | **EXTREMELY** | | |
| 1. Felt bitter or angry about the loss? | **NOT AT ALL** | | **A LITTLE** | | | | | | **QUITE A LOT** | | | **EXTREMELY** | | |
| 1. Blamed yourself a lot for the person’s death? | **NOT AT ALL** | | **A LITTLE** | | | | | | **QUITE A LOT** | | | **EXTREMELY** | | |
| 1. Tried very hard to avoid reminders of the loss (e.g., avoiding people, places, or situations associated with the person)? | **NOT AT ALL** | | **A LITTLE** | | | | | | **QUITE A LOT** | | | **EXTREMELY** | | |
| 1. Felt strong urges to be with the person? | **NOT AT ALL** | | **A LITTLE** | | | | | | **QUITE A LOT** | | | **EXTREMELY** | | |
| 1. Had difficulty trusting other people since the person’s death? | **NOT AT ALL** | | **A LITTLE** | | | | | | **QUITE A LOT** | | | **EXTREMELY** | | |
| 1. Felt alone or cut off from other people since the person’s death? | **NOT AT ALL** | | **A LITTLE** | | | | | | **QUITE A LOT** | | | **EXTREMELY** | | |
| 1. Felt that emptiness in your life or that life is meaningless without the person? | **NOT AT ALL** | | **A LITTLE** | | | | | | **QUITE A LOT** | | | **EXTREMELY** | | |
| 1. Felt unable to cope or do things you normally do without the person? | **NOT AT ALL** | | **A LITTLE** | | | | | | **QUITE A LOT** | | | **EXTREMELY** | | |
| 1. Felt confused about your role in life? | **NOT AT ALL** | | **A LITTLE** | | | | | | **QUITE A LOT** | | | **EXTREMELY** | | |
| 1. Felt that a part of you died with the person? | **NOT AT ALL** | | **A LITTLE** | | | | | | **QUITE A LOT** | | | **EXTREMELY** | | |
| 1. Had difficulty or been reluctant to plan for the future or pursuing other interests since the person’s death? | **NOT AT ALL** | | **A LITTLE** | | | | | | **QUITE A LOT** | | | **EXTREMELY** | | |
| 1. Experienced these symptoms nearly every day? | **NOT AT ALL** | | **A LITTLE** | | | | | | **QUITE A LOT** | | | **EXTREMELY** | | |
|  |  | |  | | | | | |  | | |  | | |
| 1. Have the symptoms occurred for 12 months or more? | | **YES** | **NO** | | | | |  |  |  |  |  |  |  |
| 1. Have the symptoms occurred for 6 months or more? | | YES | NO | | | | |  |  |  |  |  |  |  |
| 1. Onset: When did you start having these symptoms? [how many months ago]? | | RECORD: ________________________________ | | | | | | | | | | | | |
| 1. Overall, have these symptoms significantly affected your life and ability to do things you normally do in the past month? | | **NOT AT ALL** | | | **A LITTLE** | **QUITE A LOT** | | | | **EXTREMELY** | | | | |

| **Adaptive Stress Index** | | | | |
| --- | --- | --- | --- | --- |
| DOMAIN 1: SAFETY/SECURITY | | | | |
| **In the past 12 months, have you:** | **NOT AT ALL** | **A LITTLE** | **QUITE A LOT** | **EXTREMELY** |
| 1. Felt very uneasy when seeing uniformed police? | **0** | **1** | **2** | **3** |
| 1. Felt very insecure in my current surroundings because of ongoing threats (e.g., arrest, repatriation)? | **0** | **1** | **2** | **3** |
| 1. Had serious concerns about whether my family will survive? | **0** | **1** | **2** | **3** |
| 1. Felt very unsafe when going out alone? | **0** | **1** | **2** | **3** |
| 1. Had serious concerns about my family might die of hunger? | **0** | **1** | **2** | **3** |
| 1. Felt very worried that the future will be insecure? | **0** | **1** | **2** | **3** |
| 1. Felt very worried about being put in the same dangerous situation (e.g. fear of returning home) as before? | **0** | **1** | **2** | **3** |
| 1. Felt very nervous about family members getting sick because we cannot afford medical care? | **0** | **1** | **2** | **3** |
| 1. Felt very worried about being evicted or forced to move elsewhere (e.g. detention center) where it will be unsafe? | **0** | **1** | **2** | **3** |
| 1. Felt very worried about the physical safety (e.g. physical assaults by others) of myself and my family? | **0** | **1** | **2** | **3** |
| 1. Felt very unsafe because of being stopped and extorted by police? | **0** | **1** | **2** | **3** |
| 1. Felt very unsafe because of being threatened by gangsters in the neighbouring? | **0** | **1** | **2** | **3** |

| DOMAIN 2: BONDS/NETWORKS | | | | |
| --- | --- | --- | --- | --- |
| **In the past 12 months, have you:** | **NOT AT ALL** | **A LITTLE** | **QUITE A LOT** | **EXTREMELY** |
| 1. Thought a lot about family members or close others who are dead or missing. | **0** | **1** | **2** | **3** |
| 1. felt very upset about having to leave behind family in Arakan or refugee camps in Bangladesh | **0** | **1** | **2** | **3** |
| 1. Felt “homesick”. | **0** | **1** | **2** | **3** |
| 1. Worried a lot about the safety of family in other places. | **0** | **1** | **2** | **3** |
| 1. Had strong urges to return to Arakan | **0** | **1** | **2** | **3** |
| 1. felt lonely without family and friends | **0** | **1** | **2** | **3** |
| 1. Felt very upset about not being able to return to Arakan | **0** | **1** | **2** | **3** |
| 1. Felt very upset about not being able to enshroud (“Kafan”) and give funeral prayers (“Janazah”) to deceased family member(s) a proper burial ceremony. | **0** | **1** | **2** | **3** |
| 1. Felt very upset because I could not carry out traditional activities (e.g. Kulkhani, give prayers at the graveyard) for deceased family members. | **0** | **1** | **2** | **3** |
| 1. Very concerned that I cannot visit the graves of my family. | **0** | **1** | **2** | **3** |
| 1. Worried a lot about my family members who have gone missing. | **0** | **1** | **2** | **3** |
| 1. Felt upset because I am separated from my community and neighbours. | **0** | **1** | **2** | **3** |
| 1. Felt upset because I could not pay tribute to the death of family members. | **0** | **1** | **2** | **3** |
| 1. Felt upset by the death of family members and friends who were part of the struggle for independence. | **0** | **1** | **2** | **3** |
| DOMAIN 3: ACCESS TO JUSTICE | | | | |
| **In the past 12 months, have you:** | **NOT AT ALL** | **A LITTLE** | **QUITE A LOT** | **EXTREMELY** |
| 1. Had strong feelings of unfairness about the way I or my family had been treated in the past? | **0** | **1** | **2** | **3** |
| 1. Thought a lot about the unjust things that happened to me and my family in the past? | **0** | **1** | **2** | **3** |
| 1. Thought a lot about the unjust things that happened to people in my community? | **0** | **1** | **2** | **3** |
| 1. Felt strong urges to make those who treated me and my family badly pay for what they did? | **0** | **1** | **2** | **3** |
| 1. Lost trust in people because of the unjust things that happened to me and my family in the past? | **0** | **1** | **2** | **3** |
| 1. Become suspicious of authorities (e.g. police) because of the unjust things that happened to me and my family in the past? | **0** | **1** | **2** | **3** |
| 1. Felt frustrated because I could not stop the unjust things from happening to my family or friends in the past? | **0** | **1** | **2** | **3** |
| 1. Noticed that I have started to look at things and people differently (e.g. not trusting people) than I did in the past? | **0** | **1** | **2** | **3** |
| 1. Had trouble forgiving those who hurt me and my family badly in the past? | **0** | **1** | **2** | **3** |
| 1. Felt frustrated because the people who hurt me and my family have not been punished? | **0** | **1** | **2** | **3** |
| 1. Had difficulty accepting the unjust things that happened to me and my family? | **0** | **1** | **2** | **3** |
| 1. Had difficulty accepting the unjust things that happened to my community? | **0** | **1** | **2** | **3** |
| 1. Had feelings of injustice because you are an asylum seeker or refugee (e.g., the way you are treated by other people)? | **0** | **1** | **2** | **3** |

| DOMAIN 4: ROLES/IDENTITIES | | | | |
| --- | --- | --- | --- | --- |
| **In the past 12 months, have you:** | **NOT AT ALL** | **A LITTLE** | **QUITE A LOT** | **EXTREMELY** |
| 1. Felt frustrated because I could not fulfil the roles (e.g. professional, student, husband, wife, son, and daughter) that I used to do? | **0** | **1** | **2** | **3** |
| 1. Felt confused about what to do with my life now and in the future it is not clear what my role is? | **0** | **1** | **2** | **3** |
| 1. Lost a sense of direction in my life? | **0** | **1** | **2** | **3** |
| 1. Felt uncertain about my future roles and responsibilities? | **0** | **1** | **2** | **3** |
| 1. Had difficulty setting goals about what I will be or do in the future because of the challenges I face as a Rohingya and because of my political beliefs? | **0** | **1** | **2** | **3** |
| 1. Felt frustrated because of the communication difficulties I experience with family members or others? | **0** | **1** | **2** | **3** |
| 1. Find the changes in my opportunities (in work, profession, society) difficult to deal with? | **0** | **1** | **2** | **3** |
| 1. Felt frustrated because I am not able to participate in festive activities (e.g. wedding ceremonies, celebrations) or attend mosques (to offer my prayers)in thesocietyin the way I used to? | **0** | **1** | **2** | **3** |
| 1. Had difficulty trying to overcome cultural barriers (e.g. wedding ceremonies, religious teaching, recitals from the Quran) so that I can have a place in society? | **0** | **1** | **2** | **3** |
| 1. Felt frustrated because I have to rely on others and cannot get things done on my own? | **0** | **1** | **2** | **3** |
| 1. Lost a sense of autonomy and control in my life? | **0** | **1** | **2** | **3** |

| DOMAIN 5: EXISTENTIAL MEANING | | | | |
| --- | --- | --- | --- | --- |
| **In the past 12 months, have you:** | **NOT AT ALL** | **A LITTLE** | **QUITE A LOT** | **EXTREMELY** |
| 1. Felt separated from the traditional way of life? | **0** | **1** | **2** | **3** |
| 1. Felt separated from activities that allow me to pursue my cultural, religious, or spiritual beliefs? | **0** | **1** | **2** | **3** |
| 1. Lost faith in religious or spiritual faith? | **0** | **1** | **2** | **3** |
| 1. Had trouble trying to adapt to customs and values in the new environment? | **0** | **1** | **2** | **3** |
| 1. Felt frustrated because I am not able to express my political aspirations in public because of government | **0** | **1** | **2** | **3** |
| 1. Felt frustrated because there is limited prospect for my life in future? | **0** | **1** | **2** | **3** |
| 1. Felt lost, as if I have lost control of my life? | **0** | **1** | **2** | **3** |
| 1. Lost a sense of purpose or meaning in my life? | **0** | **1** | **2** | **3** |
| 1. Had trouble making sense of the bad things that happened in my life? | **0** | **1** | **2** | **3** |
| 1. Felt frustrated because I could not have access to religious or spiritual practices? | **0** | **1** | **2** | **3** |
| 1. Felt separated from my family traditions, values, and beliefs? | **0** | **1** | **2** | **3** |
| 1. Felt worried about the future of my country day? | **0** | **1** | **2** | **3** |
| 1. Felt worried about the strong influence of MILITARY JUNTA and the 969 group in Arakan? | **0** | **1** | **2** | **3** |
| 1. Felt that the upheavals I have been through have changed my belief in life and humanity. | **0** | **1** | **2** | **3** |

| **RESILIENCE SCALE** | | | | | |
| --- | --- | --- | --- | --- | --- |
| For each item, please mark an "x" in the box below that best indicates how much you agree with the following statements as they apply to you over the last month. If a particular situation has not occurred recently, answer according to how you think you would have felt. | | | | | |
|  | **Not true at all [0]** | **Rarely true [1]** | **Sometimes true [2]** | **Often true [3]** | **True nearly all the time [4]** |
| 1. I am able to adapt when changes occur. | **0** | **1** | **2** | **3** | **4** |
| 1. I have at least one close and secure relationship that helps me when I am stressed. | **0** | **1** | **2** | **3** | **4** |
| 1. When there are no clear solutions to my problems, sometimes fate or God can help. | **0** | **1** | **2** | **3** | **4** |
| 1. I can deal with whatever comes my way. | **0** | **1** | **2** | **3** | **4** |
| 1. Past successes give me confidence in dealing with new challenges and difficulties. | **0** | **1** | **2** | **3** | **4** |
| 1. I try to see the humorous side of things when I am faced with problems. | **0** | **1** | **2** | **3** | **4** |
| 1. Havi ng to cope with stress can make me stronger. | **0** | **1** | **2** | **3** | **4** |
| 1. I ten d to bounce back after illness, injury, or other hardships. | **0** | **1** | **2** | **3** | **4** |
| 1. Goo d or bad, I believe that most things happen for a reason. | **0** | **1** | **2** | **3** | **4** |
| 1. I give my best effort no matter what the outcome may be | **0** | **1** | **2** | **3** | **4** |
| 1. I bel ieve I can achieve my goals, even if there are obstacles. | **0** | **1** | **2** | **3** | **4** |
| 1. Even when things look hopeless, I don't give up. | **0** | **1** | **2** | **3** | **4** |
| 1. During times of stress/crisis, I know where to turn for help. | **0** | **1** | **2** | **3** | **4** |
| 1. Under pressure, I stay focused and think clearly. | **0** | **1** | **2** | **3** | **4** |
| 1. I pre fer to take the lead in solving problems rather than letting others make all the decisions. | **0** | **1** | **2** | **3** | **4** |
| 1. I am not easily discouraged by failure. | **0** | **1** | **2** | **3** | **4** |
| 1. I think of myself as a strong person when dealing with life's challenges and difficulties. | **0** | **1** | **2** | **3** | **4** |
| 1. I can make unpopular or difficult decisions that affect other people, if it is necessary. | **0** | **1** | **2** | **3** | **4** |
| 1. I am able to handle unpleasant or painful feelings like sadness, fear, and anger. | **0** | **1** | **2** | **3** | **4** |
| 1. In dealing with life's problems, sometimes you have to act on a hunch without knowing why. | **0** | **1** | **2** | **3** | **4** |
| 1. I have a strong sense of purpose in life. | **0** | **1** | **2** | **3** | **4** |
| 1. I fee l in control of my life. | **0** | **1** | **2** | **3** | **4** |
| 1. I like challenges. | **0** | **1** | **2** | **3** | **4** |

**SCORING GUIDE**

**1. MENTAL HEALTH PROBLEMS**

***A. DEPRESSION***

**ANY 3 or 4 OF THE FOLLOWING**

*YES= A LITTLE/QUITE A LOT/EXTREMELY*

MDD 3 [YES | NO]

MDD 4 [YES | NO]

MDD 5 [YES | NO]

MDD 6 [YES | NO]

MDD 7 [YES | NO]

MDD 8 [YES | NO]

MDD 9 [YES | NO]

MDD 10 [YES | NO]

NUMBER OF ITEMS ANSWERED YES: ______

**AND**

MDD 1 [YES | NO]

**OR**

MDD 2 [YES | NO]

NUMBER OF ITEMS ANSWERED YES: ______ AT LEAST 1 OR 2 ANSWERED YES?

TOTAL NUMBER OF ITEMS YES: ____ **AT LEAST 5** ANSWERED YES **INLCLUDING EITHER MDD 1 OR MDD 2?**

***MET CRITERIA FOR DEPRESSION:***

***B. PTSD/COMPLEX PTSD***


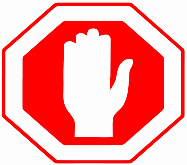


[This Photo](http://en.wikipedia.org/wiki/File:Israeli_Stop_Sign.png) by Unknown Author is licensed under [CC BY-SA](https://creativecommons.org/licenses/by-sa/3.0/)

**AT LEAST 1 TRAUMATIC EVENT?**

**YES | NO [IF NO, SKIP]**

**I. ANY 1 OF THE FOLLOWING**

*YES= A LITTLE/QUITE A LOT/EXTREMELY*

| PTSD 1 [YES \| NO] |
| --- |
| PTSD 2 [YES \| NO] |
| PTSD 3 [YES \| NO] |
| PTSD 4 [YES \| NO] |
| PTSD 5 [YES \| NO] |

NUMBER OF ITEMS ANSWERED YES: ______ AT LEAST 1?

**AND**

**II.** **ANY 1 OF THE FOLLOWING**

| PTSD 6 [YES \| NO] |
| --- |
| PTSD 7 [YES \| NO] |

NUMBER OF ITEMS ANSWERED YES: _______ AT LEAST 1?

**AND**

**III. ANY 2 OF THE FOLLOWING**

| PTSD 8 [YES \| NO] | PTSD 14 [YES \| NO] |
| --- | --- |
| PTSD 9 [YES \| NO] | PTSD 15 [YES \| NO] |
| PTSD 10 [YES \| NO] |  |
| PTSD 11 [YES \| NO] |  |
| PTSD 12 [YES \| NO] |  |
| PTSD 13 [YES \| NO] |  |

NUMBER OF ITEMS ANSWERED YES: ______ AT LEAST 2?

**AND**

**IV. ANY 2 OF THE FOLLOWING**

| PTSD 16 [YES \| NO] |
| --- |
| PTSD 17 [YES \| NO] |
| PTSD 18 [YES \| NO] |
| PTSD 19 [YES \| NO] |
| PTSD 20 [YES \| NO] |
| PTSD 21 [YES \| NO] |

NUMBER OF ITEMS ANSWERED YES: ______ AT LEAST 2?

TOTAL NUMBER OF ITEMS YES: ____ **AT LEAST 6** ANSWERED YES **INLCLUDING 1 OF I, II AND 2 OF III, IV?**

**B. 1. SCREENING FOR COMPLEX PTSD**


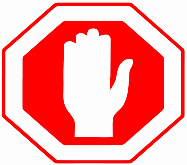


[This Photo](http://en.wikipedia.org/wiki/File:Israeli_Stop_Sign.png) by Unknown Author is licensed under [CC BY-SA](https://creativecommons.org/licenses/by-sa/3.0/)

**AT LEAST 2 OR MORE TRAUMATIC EVENTS?**

**YES | NO (IF NO, SKIP)**

*YES= A LITTLE/QUITE A LOT/EXTREMELY*

1. PTSD 2 **OR** PTSD 3 = YES / NO
2. PTSD 6 **OR** PTSD 7 = YES / NO
3. PTSD 9 **OR** PTSD 10 = YES / NO
4. PTSD 11 **OR** PTSD 16 = YES / NO
5. PTSD 13 **OR** PTSD 14 = YES / NO
6. PTSD 18 **OR** PTSD 19 = YES / NO

TOTAL NUMBER OF ITEMS YES: ____ **AT LEAST 6** ANSWERED YES **INLCLUDING 1 OF I, II, III, IV, V, VI?**

***MET CRITERIA FOR PTSD:***

***MET CRITERIA FOR COMPLEX PTSD:***

***C. GENERALIZED ANXIETY DISORDER***


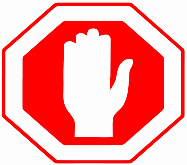


[This Photo](http://en.wikipedia.org/wiki/File:Israeli_Stop_Sign.png) by Unknown Author is licensed under [CC BY-SA](https://creativecommons.org/licenses/by-sa/3.0/)

**AT LEAST 1 ONGOING WORRY (E.G. HOUSING, FOOD, INCOME, ETC)**

**GAD A1—GAD A7**

**YES | NO (IF NO, SKIP)**

*YES= A LITTLE/QUITE A LOT/EXTREMELY*

**GAD A8 = YES | NO**

**ANY 3 OF THE FOLLOWING**

GAD 1 [YES | NO]

GAD 2 [YES | NO]

GAD 3 [YES | NO]

GAD 4 [YES | NO]

GAD 5 [YES | NO]

GAD 6 [YES | NO]

TOTAL NUMBER OF ITEMS YES: ____ **AT LEAST 3** ANSWERED YES (GAD 1—GAD 6) **INLCLUDING GAD A8 + AT LEAST 1 OF GAD A1—GAD A7**

***MET CRITERIA FOR GAD:***

**2. ADAPTIVE DISTRESS (ADAPTIVE STRESS INDEX/ASI)**

**1 OR MORE ITEMS** ANSWERED **YES (ANSWER: 1—3)** OF

**SAFETY/SECURITY [YES | NO]**

1. **1 OR MORE ITEMS** ANSWERED **YES** **(ANSWER: 1—3)** OF

**BONDS/ATTACHMENTS [YES | NO]**

1. **1 OR MORE ITEMS** ANSWERED **YES** **(ANSWER: 1—3)** OF

**JUSTICE [YES | NO]**

1. **1 OR MORE ITEMS** ANSWERED **YES** **(ANSWER: 1—3)** OF

**ROLES/IDENTITIES [YES | NO]**

1. **1 OR MORE ITEMS** ANSWERED **YES** **(ANSWER: 1—3)** OF

**MEANING [YES | NO]**

**AT LEAST 1 OR MORE FOR A, B, C, D, E ABOVE:**

**YES | NO**

**TRIAL ELIGBILITY**

**MET CRITERIA FOR AT LEAST 1 OF:**

1. **DEPRSSION**
2. **PTSD**
3. **COMPLEX**
4. **GAD**

**YES | NO**

**AND**

**MET CRITERIA FOR FIVE DOMAINS OF ADAPTIVE STRESS:**

**YES | NO**

**TRIAL ELIGBLE
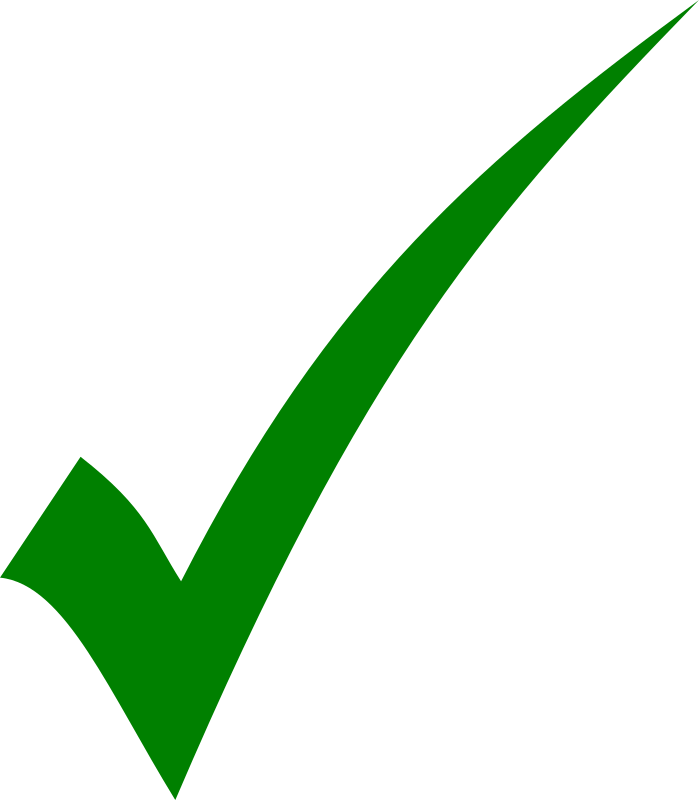
 TRIAL NOT ELIGBLE
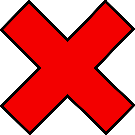
**
